# Hepatocellular carcinoma cells loss lenvatinib efficacy in vitro through autophagy and hypoxia response-derived neuropilin-1 degradation

**DOI:** 10.1038/s41401-022-01021-2

**Published:** 2022-11-14

**Authors:** Paula Fernández-Palanca, Tania Payo-Serafín, Beatriz San-Miguel, Carolina Méndez-Blanco, María J. Tuñón, Javier González-Gallego, José L. Mauriz

**Affiliations:** 1 Institute of Biomedicine (IBIOMED), University of León, Campus de Vegazana s/n, 24071 León, Spain; 2 Centro de Investigación Biomédica en Red de Enfermedades Hepáticas y Digestivas (CIBERehd), Instituto de Salud Carlos III, Av. de Monforte de Lemos, 5, 28029 Madrid, Spain

**Keywords:** hepatocellular carcinoma cells, neuropilin-1, autophagy, hypoxia, HIF-1α, lenvatinib

## Abstract

Despite pharmacological advances such as lenvatinib approval, therapeutic failure of hepatocellular carcinoma (HCC) remains a big challenge due to the complexity of its underlying molecular mechanisms. Neuropilin-1 (NRP1) is a co-receptor involved in several cellular processes associated to chemoresistance development. Since both the double-edged process of autophagy and hypoxia-derived response play crucial roles in the loss of therapeutic effectiveness, herein we investigated the interplay among NRP1, autophagy and hypoxia in development of lenvatinib resistance in HCC cell lines. We first analyzed NRP1 expression levels in human HCC samples from public databases, found significantly increased NRP1 expression in human HCC samples as well as its correlation with advanced tumor and metastasis stages. Among 3 HCC cell lines (HepG2, Huh-7 and Hep3B), Hep3B and Huh-7 cells showed significantly increased NRP1 expression levels and cell migration ability together with higher susceptibility to lenvatinib. We demonstrated that NRP1 gene silencing significantly enhanced the anticancer effects of lenvatinib on Hep3B and Huh-7 cells. Furthermore, lenvatinib suppressed NRP1 expression through promoting autophagy in Hep3B and Huh-7 cells; co-treatment with bafilomycin A1 attenuated the antitumor effects of lenvatinib, and NRP1 silencing prevented this loss of in vitro effectiveness of lenvatinib even in the presence of bafilomycin A1. In addition, exposure to a hypoxic microenvironment significantly decreased NRP1 expression through autophagy in Hep3B and Huh-7 cells. Under hypoxia, HIF-1α directly modulated NRP1 expression; HIF-1α silencing not only enhanced the anticancer effects of combined lenvatinib and hypoxia, but also prevented the loss of effectiveness caused by bafilomycin A1, highlighting the potential role of HIF-1α-derived hypoxia response in the adaptive cellular response to lenvatinib and promoting resistance acquisition by autophagy modulation. Overall, NRP1 may constitute a potential therapeutic target to prevent lenvatinib failure derived from a hypoxia-associated modulation of autophagy in advanced HCC.

## Introduction

Hepatocellular carcinoma (HCC) is the most frequent type of liver cancer and is placed as the third cause of cancer-related death worldwide, after lung and colorectal cancers [1]. HCC constitutes 80%-90% cases of primary liver cancer and remains a threatening health problem [1, 2]. However, despite novel findings in tumor therapy have been obtained in recent years and the emerging targets, therapeutic failure is still an important challenge that needs to be addressed [3].

Even though the tyrosine kinase inhibitor (TKI) sorafenib was the only drug approved against advanced HCC for a decade, lenvatinib antitumor properties have shown an increased median overall survival along with better second outcomes in HCC patients, becoming the first therapeutic alternative to sorafenib [2]. TKIs are molecular targeted drugs that specifically inhibit cellular proteins involved in key processes that modulate tumor progression such as cell proliferation, apoptosis, angiogenesis and cell migration [3]. Nonetheless, HCC cells have proved to develop resistance mechanisms that overcome antitumor actions of these drugs, leading to tumor progression [2–4]. Among these mechanisms, different cellular and molecular processes have been reported to contribute to resistance acquisition, including apoptosis evasion, autophagy, hypoxia-derived response, activation of oncogenic pathways, angiogenesis, metastasis and microRNAs [4–6].

Neuropilin-1 (NRP1) is a transmembrane glycoprotein which acts as a co-receptor of semaphorins (SEMAs), axon guidance molecules, and different growth factors involved in key oncogenic signaling pathways [7, 8]. NRP1 is a non-tyrosine kinase receptor that interacts with vascular endothelial growth factor (VEGF)/VEGF receptor (VEGFR), placenta growth factor (PlGF)/VEGFR, hepatocyte growth factor (HGF)/HGF receptor (cMet), and transforming growth factor β1 (TGFβ1)/TGFβ receptor (TGFβR), among others [7, 9]. Although NRP1 mainly modulates axon guidance and angiogenesis processes, recent evidence highlights the potential role of NRP1 in tumor progression and therapeutic failure, arising as a potential prognostic biomarker and therapeutic target [7–9]. Several investigations have revealed that NRP1 may be a crucial modulator of angiogenesis and cell migration, key processes involved in the loss of chemosensitivity in cancer, mostly to antiangiogenic drugs [7, 9]. In this line, NRP1 has emerged as an interesting mediator protein in the loss of drug sensitivity, probably involved in the activation of alternative pathways during the development of drug chemoresistance [8]. Nonetheless, loss of therapeutic effectiveness is a complex process in which numerous mechanisms that need to be fully understood are involved [4, 5].

Hypoxia conditions have been described to be a relevant process in resistance acquisition in HCC [4, 5, 10]. The adaptive response to hypoxia is mainly modulated by the hypoxia-inducible factors 1α (HIF-1α) and 2α (HIF-2α). These proteins are transcription factors of multiple proteins involved in glycolysis, cell cycle, angiogenesis, mitophagy and multi-drug resistance (MDR) pathways [5, 6]. While HIF-2α mediates chronic hypoxia response, HIF-1α drives the response to acute hypoxia, playing an essential role in cell adaptation to hypoxic microenvironment [5, 6]. Recent investigations have focused on the potential role of HIFs in tumor progression and chemoresistance development [11, 12]. The anti-angiogenic effects of TKIs, such as sorafenib or lenvatinib, lead to hypoxia induction in the tumoral tissue, driving to an adaptive cell response to drug treatment mediated by the HIFs [5, 6]. This close relationship between hypoxia and TKIs has recently become the focus of interest in the cancer research field, especially in HCC [11–13].

Although numerous processes are involved in the loss of drug sensitivity in HCC [4, 10], autophagy arouses a distinct interest because of the dual role this mechanism has on cancer development and progression [14, 15]. Autophagy is a highly conserved self-degradative process that maintains cellular homeostasis [14, 16], which has been associated with both antitumoral and pro-survival effects, as well as to increase drug effectiveness or promote drug resistance in HCC [16, 17]. Due to the complexity that underlies therapeutic failure, further studies that allow a deeper understanding to improve the treatment landscape of advanced HCC are required.

In the current study, we evaluated the role of NRP1 on the loss of therapeutic effectiveness of lenvatinib and the modulation exerted by the HIF-1α-mediated response to hypoxia. We determined the potential interest of NRP1 as a molecular target preventing autophagy-associated resistance development in an in vitro model of human HCC complemented with human HCC patient data from available databases.

## Materials and methods

### Gene expression analysis in human HCC samples

The gene expression levels of NRP1 in HCC patients were obtained from different public databases, the Human Protein Atlas (HPA) (https://www.proteinatlas.org/) [18] and UALCAN (http://ualcan.path.uab.edu/analysis.html) [19], Gene Expression Profiling Interactive Analysis (GEPIA) (http://gepia.cancer-pku.cn/) [20], and University of California Santa Cruz (UCSC) Xena (https://xenabrowser.net/heatmap/) [21], performing data analysis and graphical representation with the tools provided by the databases. Moreover, RNA-seq data from tumor and paired non-tumor tissue from human HCC patients were obtained from Gene Expression Omnibus (GEO) database (https://www.ncbi.nlm.nih.gov/geo/) [22], accession GSE14520 [23]. In total, 1039 human HCC samples and 662 human liver samples from four different dataset sources were analyzed, The Cancer Genome Atlas (TCGA), Genomic Data Commons (GDC)-TCGA, Genotype-Tissue Expression (GTEx) and GSE14520 datasets.

### Gene correlation analysis of NRP1 in HCC patient samples

The presence of gene expression correlation between NRP1 and autophagy-related genes was analyzed in the UALCAN database (http://ualcan.path.uab.edu/analysis.html) [19]. We identified potential genes from the autophagy KEGG pathway (hsa04140), which encode for proteins involved in all the steps of the autophagy process. Then, we searched for the positive or negative correlation between NRP1 and genes from this autophagy pathway, determining the Pearson-coefficient correlation (Pearson-CC) values.

For correlation analysis between NRP1 and HIF-1α, we employed the above-mentioned UALCAN database (http://ualcan.path.uab.edu/analysis.html) [19], as well as the UCSC Xena (https://xenabrowser.net/heatmap/) [21] and GEPIA (http://gepia.cancer-pku.cn/) [20] databases. Pearson-CC was also obtained from the three sources, evaluating the potential correlation of NRP1 with HIF-1α in two different datasets, TCGA and GDC-TCGA.

### Cell culture and reagents

Human HCC cell lines HepG2, Hep3B and Huh-7 were obtained from the American Type Culture Collection (Manassas, VA, USA) and cultured in DMEM-high glucose (Sigma-Aldrich, St Louis, MO, USA) supplemented with 10% fetal bovine serum and penicillin/streptomycin (100 U/mL, Gibco^™^, Gaithersburg, MD, USA) at 37 °C under a humidified 5% CO_2_ atmosphere during passages no longer than 25 passage. For cell treatments, lenvatinib (E7080, Selleckchem, Houston, TX, USA), first-line drug approved against advanced HCC [2], was employed at different concentrations from 0.5 to 30 µM. EG00229 trifluoroacetate was used as an antagonist of NRP1 activity, 300 µM cycloheximide (CHX) as protein synthesis inhibitor, 30 µM MG132 as proteasome inhibitor and 100 nM bafilomycin A1 as specific inhibitor of autophagy flux. All these reagents were acquired from Tocris Bioscience (Bristol, UK). Finally, CoCl_2_ (Panreac AppliChem, Barcelona, Spain) was used as hypoximimetic at 100 µM to induce an in vitro hypoxic microenvironment.

### Acridine orange staining

Cells were seeded in 8-chamber culture slides and after corresponding treatments, they were washed with PBS, incubated for 15 min at 37 °C with 1 μg/mL acridine orange (Sigma-Aldrich) under dark conditions, removing excess dye by washing again with PBS. Briefly, samples were dried, mounted and visualized in the Nikon Eclipse E600 microscope (Nikon Instruments Inc., Melville, NY, USA). The images were analyzed with NIS-Elements (Nikon Instruments Inc.) and Fiji/ImageJ (National Institutes of Health, Bethesda, MD, USA) software. For results analysis, the corrected total cell fluorescence (CTCF) formula was used, calculating the red/green CTCF ratio relative to cell number [24].

### Immunofluorescence and laser confocal microscopy

Immunocytochemistry was conducted as previously described [25]. For overnight incubation at 4 °C we employed primary antibodies against Ki67 (1:200, sc-23900, Santa Cruz Biotechnology, Dallas, TX, USA) or NRP1 (1:250, ab81321, Abcam, Cambridge, UK). Afterwards, cells were washed thrice with PBS and incubated for 1 h at room temperature with the secondary antibodies goat anti-rabbit conjugated with Alexa Fluor®647 (1:1000, ab150079, Abcam) or goat anti-mouse conjugated with Alexa Fluor®488 (1:1000, ab150113, Abcam). Then, the coverslips were washed three times with PBS, incubated with DAPI for nucleic acid staining (Sigma-Aldrich) for 5 min at room temperature, washed again three times with PBS and mounted on glass slides with the mounting medium Fluoromount-G^TM^ (Thermo Fisher Scientific, Waltham, MA, USA). The immunocytochemistry (ICC) samples were visualized in a Zeiss LSM 800 confocal laser scanning microscope (Zeiss, Jena, Germany). The images were analyzed using Zeiss ZEN and Fiji/ImageJ software, employing the CTCF formula relative to cell number to represent results.

### Western blot

After the corresponding treatments, cells were washed with cold PBS and scraped in a homogenization buffer, following our previous protocol [11, 25]. We employed the primary antibodies HIF-1α (rabbit polyclonal IgG, 1:500, ab2185, Abcam), NRP1 (rabbit monoclonal IgG, 1:1000, ab81321, Abcam), sequestosome-1 (p62/SQSTM1) (rabbit polyclonal, 1:1000, #5114, Cell Signaling) and microtubule-associated proteins 1 A/1B light chain 3B (LC3-II) (rabbit monoclonal IgG, 1:1000, #12741, Cell Signaling), as well as the β-actin antibody (A3854, Sigma-Aldrich) as loading control. Proteins were visualized using Pierce^™^ ECL Western blotting substrate (Thermo Fisher Scientific) and densitometry reading of each protein band was performed with Fiji/ImageJ software.

### Autophagic flux assay

Autophagic flux index was calculated as the ratio of the protein expression of LC3-II when treated with bafilomycin A1 to the protein expression of LC3-II in the absence of bafilomycin A1 using results from the Western blot determination of LC3. Previously, LC3-II levels were corrected to the corresponding β-actin.

### Real-time reverse transcription polymerase chain reaction (qRT-PCR)

Gene expression analysis through qRT-PCR was conducted as previously described [11], employing the QuantStudio® 5 System qRT-PCR (Thermo Fisher Scientific). We used the human primers for NRP1 forward (5′-CGGGACCCATTCAGGATCAC-3′) and reverse (5′-CAGGTCTGCTGGTTTTGCAC-3′), and 18 S rRNA forward (5′-CCGAAGATATGCTCATGTGG-3′) and reverse (5′-TCTTGTACTGGCGTGGATTC-3′) (Sigma-Aldrich) as endogenous control. Relative changes in levels of gene expression were detected by the 2^−ΔΔCt^ method [26].

### Cell viability assays

Cell viability analyses were performed by CellTiter-Glo® Luminescent Cell Viability Assay (Promega, Madison, WI, USA) or by 3-(4,5-dimethylthiazol-2-yl)-2,5-diphenyl-tetrazolium bromide (MTT) (Sigma-Aldrich) assay as previously described [27]. For CellTiter-Glo® assay, manufacturer’s instructions were followed, measuring luminescence with the Synergy^™^ HT Multi-Mode Microplate Reader and Gen5 1.11 software. Otherwise, MTT assay was performed by removing cell media, washing with PBS and incubating cells with a 1:10 serum-free medium solution containing 5 mg/mL MTT for 3 h at 37 °C. Then, media was removed and DMSO was added to dissolve formazan crystals, shaking for 5 min under dark conditions. Finally, absorbance at 560 nm was measured with the Synergy^™^ HT Multi-Mode Microplate Reader and Gen5 1.11 software.

### Colony formation assay

Cells were harvested and subsequently seeded at 20,000 cells per well in 6-well plates to assess ability of colony formation. After cell attachment for 24 h, silencing and/or the corresponding treatments were administered, and cells were incubated for 7 days at 37 °C and 5% CO_2_. Then, cells were washed twice with PBS, fixed with 4% formaldehyde (Thermo Fisher Scientific) for 15 min and washed with Milli-Q water. Cell staining was performed by incubation with 0.1% crystal violet (Labkem, Barcelona, Spain) dissolved in 10% ethanol for 15 min. Finally, cells were washed again with Milli-Q water and air-dried. Colonies were photographed and counted with Fiji/ImageJ software.

### Cell migration assay

Cells were seeded in 6-well plates at 90% confluency and subjected to the corresponding silencing and/or treatments. Then, medium was removed and a straight scratch in the cell monolayer was performed using a sterile 200-µL pipette tip, washing twice with medium to eliminate non-adherent cells. Complete medium was added, and pictures were taken with Eclipse TE2000 inverted microscope (Nikon Instruments Inc.) at 0, 4, 8, 12 and 24 h for initial migration analysis in HepG2, Hep3B and Huh-7 cell lines, and at 0 h and 24 h for the rest of experiments. Images were analyzed with Fiji/ImageJ software. Cell migration ability was measured by calculating percentage of wound closure based on the formula: ([(wound area at 0 h-wound area at time point)/(wound area at 0 h)] × 100) [28].

### Gene silencing

For specific gene silencing, cells were initially seeded in 6-well plates and, after 24 h, ON-TARGETplus Human HIF1A siRNA SMARTPool (a mixture of 4 siRNA targeting HIF1A), ON-TARGETplus Human NRP1 siRNA SMARTPool (a mixture of 4 siRNA targeting NRP1) or ON-TARGETplus Non-targeting Control Pool (a negative control pool of 4 siRNA) were introduced into cells using the DharmaFECT 4 Transfection Reagent (Horizon Discovery, Waterbeach, UK) and serum-reduced Opti-MEM^™^ medium (Gibco^™^, Thermo Fisher Scientific) following the manufacturer’s protocol. At 8 h post-transfection, medium was replaced by complete fresh DMEM-high glucose medium and cells were re-seeded according to the different experiments. After 24 h from transfection, treatments were performed and maintained during 24 h, and the corresponding assays were performed 48 h post-transfection and 24 h post-treatments.

### Statistical analysis

The statistical *P*-values from human data analysis were provided directly for the corresponding databases, regardless of GEO database and the GSE14520 dataset, in which NRP1 overexpression was analyzed and represented with the software GraphPad Prism 8. Likewise, correlation significance between NRP1 and the above-mentioned genes in human HCC samples, together with the in vitro results (represented as mean values ± SD), were analyzed with the statistical package GraphPad Prism 8 (GraphPad Software, San Diego, CA, USA). For each corresponding experiment, unpaired *t-*test, one-way or two-way ANOVA followed by Tukey, Dunnett or Sidak post-hoc tests were used. Statistical significance was considered when *P* < 0.05.

## Results

### NRP1 is overexpressed in human HCC tissue and is correlated with advanced stages and nodal metastasis status

**Fig. 1:**
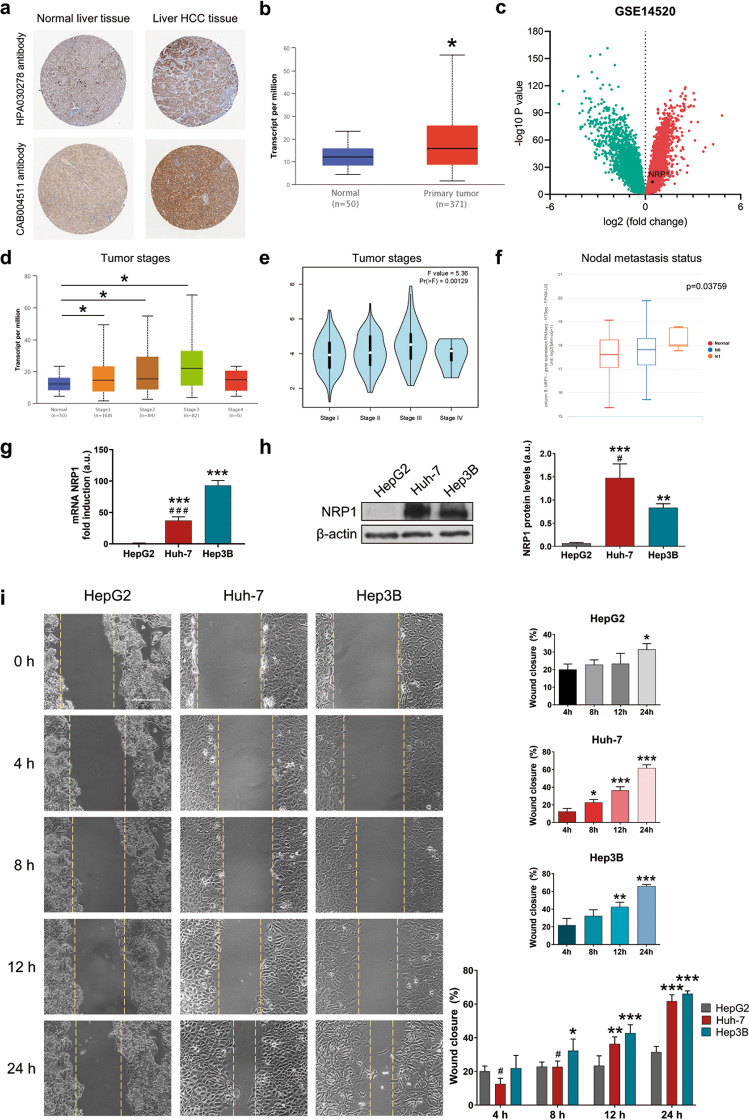
Characterization of NRP1 expression in human samples and HCC cell lines, and cell migration ability. Representative images of NRP1 immunohistochemistry in normal liver and HCC tissues from the (**a**) HPA database and comparative analysis of NRP1 expression levels obtained from the (**b**) UALCAN database employing the TCGA gene datasets. Identification of significantly downregulated (green) or upregulated (red) expressed genes in HCC GSE14520 dataset from the (**c**) GEO database. Association of NRP1 levels with tumor stages from the (**d**) UALCAN and (**e**) GEPIA databases, and with different nodal metastasis status from the (**f**) UCSC Xena database. Significant differences when *P < 0.05. Comparison of the (**g**) mRNA and (**h**) protein levels of NRP1 determined in the three HCC cell lines HepG2, Huh-7 and Hep3B by qRT-PCR and Western blot, respectively. A representative immunoblot for each protein with the quantification of the corresponding triplicates is shown. Data are expressed as mean values of arbitrary units (a.u.) ± SD (*n* = 3). *P < 0.05, **P < 0.01, ***P < 0.001 *vs* HepG2; ^#^*P* < 0.05 *vs* Hep3B. **i** Cell migration ability was evaluated by wound-healing assay, representing the % of the wound closure area after 4, 8, 12 and 24 h from the scratch, separately for each cell line and comparing the three HCC lines. Magnification 10×, scale bar 50 µm. For each cell line analysis *P < 0.05, **P < 0.01, ***P < 0.001 *vs* 4 h. For comparison analysis of the three cell lines *P < 0.05, **P < 0.01, ***P < 0.001 *vs* HepG2; ^#^*P* < 0.05 *vs* Hep3B.

To determine the potential interest of NRP1 in the development and progression of HCC, we analyzed the NRP1 expression levels in different HCC datasets from human databases (Fig. 1a–f). Representative images of NRP1 immunohistochemistry in normal liver and HCC tissue of the TCGA dataset were obtained from the HPA database with both HPA030278 and CAB004511 antibodies, observing a strong NRP1 staining in the liver HCC tissue (Fig. 1a). Through UALCAN database, a significantly increased NRP1 expression was identified in the primary tumor tissue of HCC samples compared to healthy normal tissue from the TCGA (Fig. 1b). It was also confirmed by an independent analysis performed with the GSE14520 dataset, in which differential-expressed genes were determined, identifying a significant overexpression of NRP1 in tumor tissues compared to paired non-tumor tissues of HCC patients (Fig. 1c).

Association of NRP1 expression levels with tumor stages in human HCC was also evaluated, finding a significant increased expression of NRP1 in advanced HCC stages from UALCAN and GEPIA databases (Fig. 1d, e). Interestingly, when this analysis was conducted in the metastatic nodal status, NRP1 was found to be overexpressed in advanced stages of nodal metastasis in human samples from the UCSC Xena database (Fig. 1f).

### The Hep3B and Huh-7 HCC cell lines showed an increased NRP1 expression and cell migration ability together with higher susceptibility to lenvatinib

To confirm the results obtained from the clinical databases and select a suitable in vitro model, we analyzed the NRP1 expression and the migration ability of different HCC cell lines (Fig. 1g, i), with different phenotypic and genotypic characteristics in accordance to the high heterogeneity of human HCC. Results showed markedly higher levels of both mRNA and protein NRP1 in Hep3B and Huh-7 in comparison to HepG2 (Fig. 1g, h). After thorough analysis of cell migration at 4, 8, 12 and 24 h, HepG2 cells exhibited the lowest wound closure ability, while both Hep3B and Huh-7 displayed greater cell migration, reaching a closure higher than 60% of the wound area at 24 h (Fig. 1i).

**Fig. 2:**
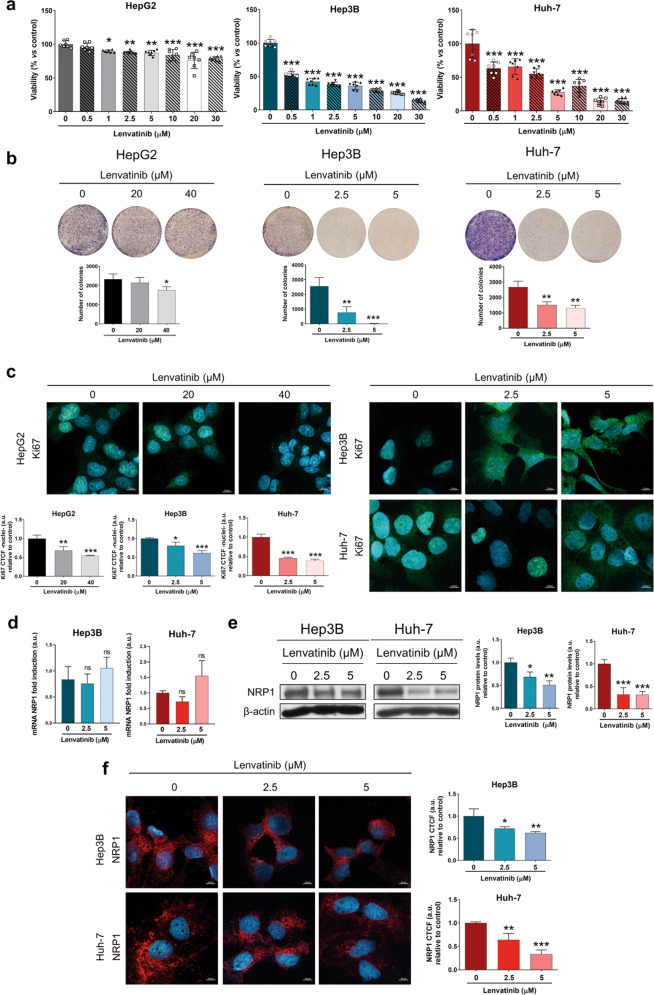
Antitumor activity of lenvatinib and modulation of NRP1 expression in the in vitro models of HCC. Different concentrations of lenvatinib ranging from 0.5 to 30 µM were used for the treatment of HCC cells during 48 h to determine the effects on (**a**) cell viability by CellTiter-Glo^®^ assay, (**b**) colony formation assay, and (**c**) nuclear translocation of Ki67 by ICC and confocal microscopy. NRP1 expression was analyzed at (**d**) mRNA levels by qRT-PCR, and at protein levels by (**e**) Western blot and (**f**) ICC after 48 h of 2.5 and 5 µM lenvatinib treatment. Data from (**a**) are represented as % of cell viability relative to non-treated cells ± SD (*n* = 7). Data from (**b**–**f**) are represented as mean values of arbitrary units (a.u.) ± SD (*n* = 3). Bar graphs from (**c**) and (**f**) represent the nuclear CTCF ratio of Ki67 and NRP1 CTCF ratio, respectively. Magnification 63×, scale bar 10 µm. **e** A representative immunoblot is shown. *P < 0.05, **P < 0.01, ***P < 0.001 and ns, not significant *vs* non-treated cells.

After identifying the potential role of NRP1 in the progression of the HCC cell lines selected, we further assessed the molecular actions derived from the lenvatinib treatment. Initially, we observed a significant inhibition of cell viability from 1 µM in HepG2, and from the lowest dose (0.5 µM) in Hep3B and Huh-7 after 48 h of lenvatinib treatment (Fig. 2a). Interestingly, HepG2 cells were less susceptible to lenvatinib than Hep3B and Huh-7 cells, not reaching a 50% cell viability inhibition; while 1 µM and 2.5 µM of lenvatinib reduced to 50% the viability of Hep3B and Huh-7, respectively (Fig. 2a). For the following analysis, the two doses of lenvatinib closest to the IC_50_ were selected, being higher for the HepG2 line. As previously observed, lenvatinib was more effective in Hep3B and Huh-7 cells, showing a significant inhibition of colony formation ability and reduction of Ki67 proliferation index with both 2.5 and 5 µM lenvatinib (Fig. 2b, c, Supplementary Fig. S1a). However, HepG2 cell line was less sensitive, colony formation was only reduced after 48 h with 40 µM lenvatinib, while nuclear localization of Ki67 was significantly decreased with 20 µM lenvatinib (Fig. 2b, c, Supplementary Fig. S1a).

Considering these findings and the close association of NRP1 and lenvatinib with cell migration and angiogenesis in cancer [9, 29], we performed the next experiments in the Hep3B and Huh-7 cell lines to precisely evaluate the role of NRP1 in lenvatinib efficacy in HCC.

### Lenvatinib diminished NRP1 protein levels in HCC cells, being responsible for the antitumor effects of lenvatinib on cell proliferation and migration

In order to elucidate the association between NRP1 and lenvatinib efficacy in the human HCC cell lines, we analyzed the effects of lenvatinib treatment on NRP1 mRNA and protein levels (Fig. 2d–f). Remarkably, a significant alteration was not observed in NRP1 mRNA levels after 48 h treatment (Fig. 2d); but, protein expression by both Western blot and ICC experienced a strong reduction when 2.5 and 5 µM lenvatinib were administered (Fig. 2e, f, Supplementary Fig. S1b).

**Fig. 3:**
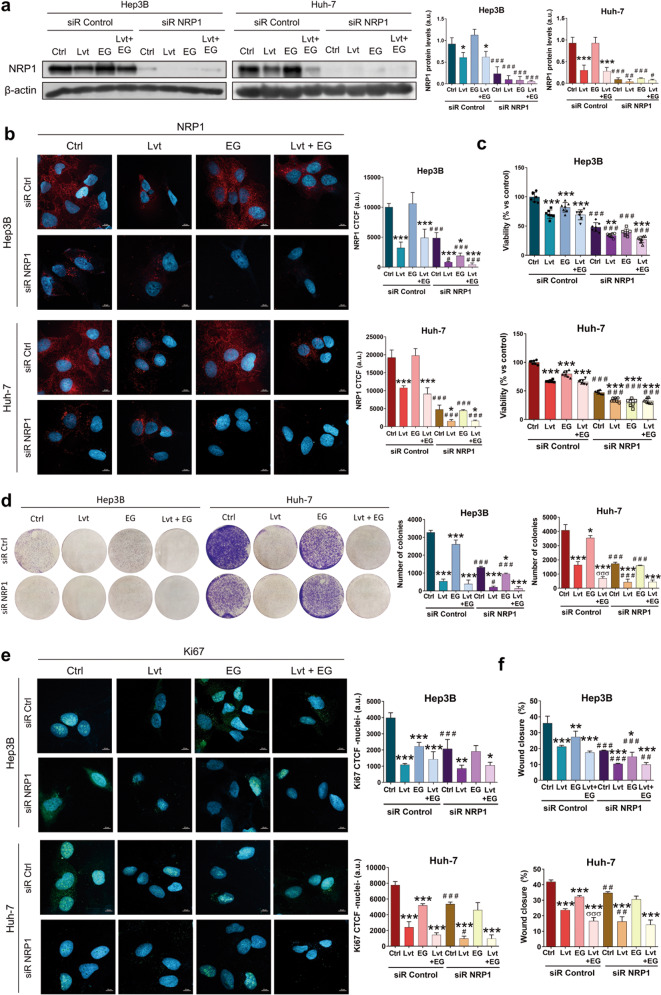
Effects of targeting NRP1 on lenvatinib actions on cell proliferation and cell migration ability. All the assays were performed 48 h post-silencing with the last 24 h of treatment with 2.5 µM lenvatinib (Lvt) and/or 15 µM EG00229 (EG). Protein levels of NRP1 were analyzed by (**a**) Western blot and (**b**) ICC, and cell viability was assessed by (**c**) CellTiter-Glo^®^, (**d**) colony formation assays, and (**e**) nuclear translocation of Ki67 by ICC and confocal microscopy. Data from (**c**) are represented as % of mean values relative to control (Ctrl) ± SD (*n* = 7). Data from (**a**), (**b**), (**d**) and (**e**) are represented as mean values of arbitrary units (a.u.) ± SD (*n* = 3). **a** A representative immunoblot is shown. Bar graphs from (**b**) and (**e**) represent the NRP1 CTCF ratio and nuclear CTCF ratio of Ki67, respectively. Magnification 63×, scale bar 10 µm. **f** Cell migration was evaluated by wound-healing assay, representing the % of the wound closure area after 24 h from the scratch. *P < 0.05, **P < 0.01, ***P < 0.001 *vs* non-treated cells in each siR group; ^#^*P* < 0.05, ^##^*P* < 0.01, ^###^*P* < 0.001 *vs* siR control; ^σσσ^*P* < 0.001 Lvt+EG *vs* Lvt treatment.

The NRP1 antagonist EG00229, hereinafter referred to as EG, acts through blockade of NRP1-VEGFA interaction, inhibiting NRP1 activity [30]. We employed EG to clarify the role of NRP1 in the antitumor actions of lenvatinib. Firstly, we selected an EG concentration by analyzing cell viability in Hep3B and Huh-7 after administration of different EG concentrations during 24 and 48 h (Supplementary Fig. S2). Although significant inhibition of cell viability was observed from the lowest dose (2.5 µM), the IC_50_ was only reached by 50 µM EG after 48 h in the Huh-7 cell line (Supplementary Fig. S2). Based on these results and published studies employing this NRP1 antagonist in cancer cells [31, 32], we selected 15 µM EG for the following experiments. In these analyses, we combined lenvatinib and EG, together with the specific gene silencing of NRP1 to assess its role in cell viability and migration in HCC (Fig. 3). Through determination of NRP1 expression by Western blot and ICC we proved that NRP1 gene silencing markedly reduced NRP1 protein levels, observing a slighter decrease in absence of gene silencing after lenvatinib administration alone and combined with EG (Fig. 3a, b, Supplementary Fig. S3a). We also observed that the individual treatment with the antagonist EG did not diminish the expression of NRP1 (Fig. 3a, b, Supplementary Fig. S3a).

The derived effects on cell viability were also evaluated through cell viability and colony formation assays, as well as the determination of the Ki67-based proliferation index. Results showed that both lenvatinib and EG significantly decreased cell viability (Fig. 3c), colony formation ability (Fig. 3d) and nuclear localization of Ki67 (Fig. 3e, Supplementary Fig. S3b) alone and combined, not displaying a synergistic effect in combination, regardless of colony formation inhibition in Huh-7 cells (Fig. 3d). However, the NRP1 silencing increased the antitumor effects in all cases in terms of cell viability (Fig. 3c) and colony formation (Fig. 3d), but only increased lenvatinib-derived effects on Ki67 proliferation index reduction in Huh-7 (Fig. 3e, Supplementary Fig. S3b).

Regarding cell migration, similar findings were also observed (Fig. 3f, Supplementary Fig. S4). The individual treatment with lenvatinib and EG significantly diminished cell migration ability, exhibiting a synergy when combined only in the Huh-7 cell line. Likewise, NRP1 gene silencing raised this migration inhibition of lenvatinib in both cell lines, but of EG only in the Hep3B cells. Interestingly, NRP1 silencing only increased the effects of lenvatinib and EG combination in Hep3B, whereas in control silenced cells a significative difference was not observed when the antagonist EG was co-administered with lenvatinib. This was not obtained in the Huh-7 cell line, in which EG did increase lenvatinib-derived inhibition of cell migration when NRP1 was silenced (Fig. 3f, Supplementary Fig. S4). Therefore, NRP1 silencing only augmented lenvatinib effects when the administration of the NRP1 antagonist EG did not achieve for increasing cell migration inhibition derived from lenvatinib.

Altogether, these results suggest that NRP1 might be mechanistically important for the antitumor effects of lenvatinib on cell proliferation and migration, but in a previous step from NRP1 activity, releasing the interest on determining the exact mechanism underlying lenvatinib actions associated with NRP1 in HCC cells.

### Lenvatinib promoted autophagy as a mechanism responsible for the NRP1 downregulation in HCC cells

**Fig. 4:**
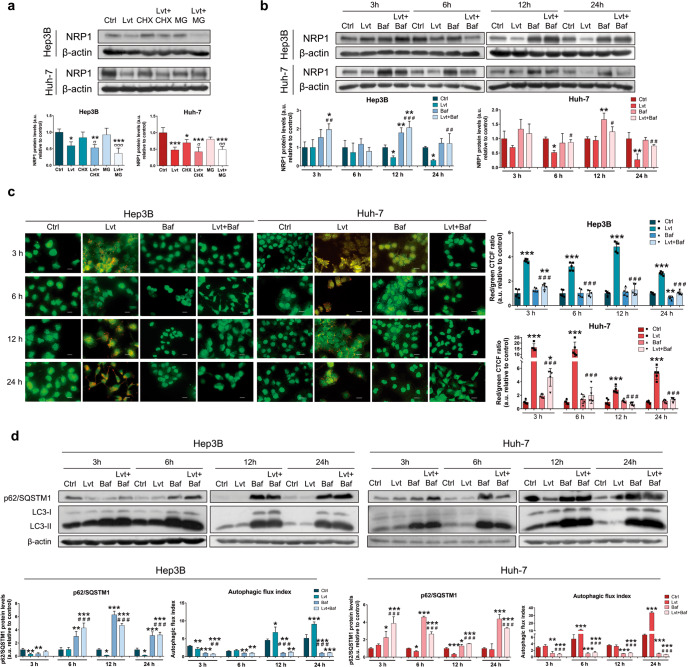
Determination of the mechanism underlying the lenvatinib-derived downregulation of NRP1. **a** Protein expression of NRP1 was analyzed by Western blot after 24 h treatment with 2.5 µM lenvatinib (Lvt) and/or 300 µM cycloheximide (CHX) or 30 µM MG132 (MG). **b** NRP1 protein levels were also determined by Western blot after 3, 6, 12 and 24 h treatment with 2.5 µM lenvatinib (Lvt) alone and combined with 100 nM bafilomycin A1 (Baf). *P < 0.05, **P < 0.01, ***P < 0.001 *vs* control; ^#^*P* < 0.05, ^##^*P* < 0.01, ^###^*P* < 0.001 combined treatment *vs* Lvt treatment; ^σ^*P* < 0.05^, σσ^*P* < 0.01, ^σσσ^*P* < 0.001 combined treatment *vs* inhibitor treatment. **c** Analysis of autolysosome cell content by acridine orange staining and fluorescence microscopy. Magnification 40×, scale bar 25 µm. Bar graphs represent the quantification of red/green CTCF ratio (*n* = 5). **d** Protein levels of p62/SQSTM1 and LC3 (LC3-I and LC3-II) were analyzed by Western blot. LC3 turnover assay was performed to determine the autophagic flux index. Data from (**a**), (**b**) and (**d**) are represented as mean values of arbitrary units (a.u.) ± SD (*n* = 3), showing a representative immunoblot. *P < 0.05, **P < 0.01, ***P < 0.001 *vs* control for each time point; ^##^*P* < 0.01, ^###^*P* < 0.001 combined treatment *vs* Lvt treatment.

To fully elucidate the exact mechanism through which lenvatinib is downregulating protein levels of NRP1, we used specific inhibitors of protein synthesis (cycloheximide, CHX, 300 µM) and protein degradation through proteasome (MG132, 30 µM) or autophagy (bafilomycin A1, 100 nM) (Fig. 4). Results exhibited that synthesis blockade reduced NRP1 protein expression; nonetheless, lenvatinib led to a higher downregulation of NRP1. Moreover, administration of the proteasome inhibitor MG132 did not alter NRP1 levels or lenvatinib effects (Fig. 4a), suggesting that inhibition of protein synthesis or induction of proteasome degradation are not the mechanisms responsible for the lenvatinib-associated reduction in NRP1 levels. Then, we employed bafilomycin A1 as a specific autophagy inhibitor of autophagosome-lysosome fusion alone and combined with lenvatinib for a time-course of 3, 6, 12 and 24 h to evaluate the dynamic process of autophagy (Fig. 4b–d). Interestingly, autophagy blockade restored the NRP1 protein levels when co-administered with lenvatinib from 12 h in Hep3B, and from 6 h in Huh-7, and this led to NRP1 protein accumulation in both HCC cell lines (Fig. 4b). In addition, the autophagy process was evaluated by acridine orange staining, showing an autophagy induction derived from lenvatinib treatment, which was significantly decreased to basal levels by bafilomycin A1 (Fig. 4c). This was also observed by protein expression analysis of the autophagic markers p62/SQSTM1 and LC3-II (Fig. 4d). Results showed an efficient blockade of lenvatinib-induced autophagy after bafilomycin A1 treatment, represented by LC3-II and p62/SQSTM1 protein accumulation, and by a significantly reduced autophagic flux index, which was also observed after bafilomycin A1 treatment alone (Fig. 4d). These results confirm the usefulness of this drug as an effective autophagy inhibitor.

Therefore, these findings indicated that autophagy may be the mechanism that underlies the NRP1 downregulation exerted by lenvatinib in the HCC cells.

### Autophagy-dependent degradation of NRP1 was the mechanism responsible for the antitumor effects of lenvatinib

**Fig. 5:**
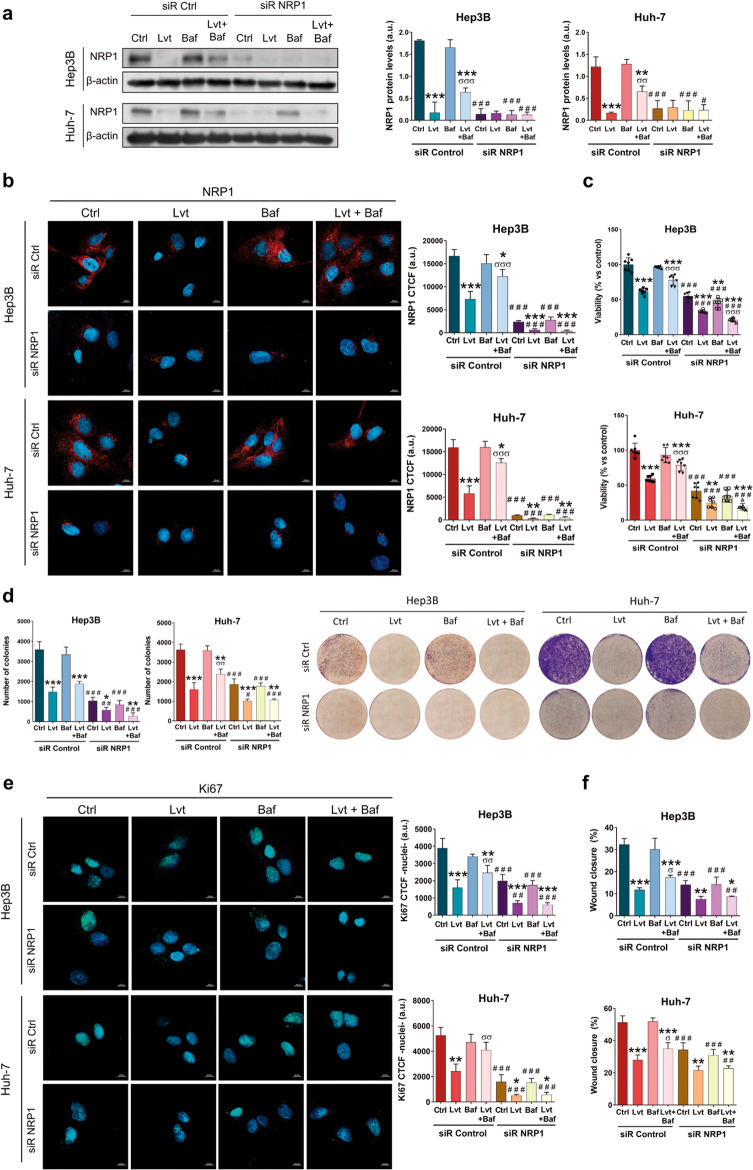
Effects derived from autophagy inhibition on NRP1 protein expression and NRP1-associated antitumor actions of lenvatinib. All the assays were performed 48 h post-silencing with the last 24 h of treatment with 2.5 µM lenvatinib (Lvt) and/or 100 nM bafilomycin A1 (Baf). Protein levels of NRP1 were analyzed by (**a**) Western blot and (**b**) ICC. Cell proliferation was assessed by (**c**) CellTiter-Glo^®^ assay, (**d**) colony formation assay, and (**e**) Ki67 proliferation index determination. Data from (**a**), (**b**), (**d**) and (**e**) are represented as mean values of arbitrary units (a.u.) ± SD (*n* = 3), showing for (**a**) a representative immunoblot. Data from (**c**) are represented as % of mean values relative to control ±SD (*n* = 7). Bar graphs from (**b**) and (**d**) represent the NRP1 CTCF ratio and the nuclear CTCF ratio of Ki67, respectively. Magnification 63×, scale bar 10 µm. **f** Cell migration ability was evaluated by wound-healing assay, representing the % of the wound closure area after 24 h from the scratch. Magnification 10× and scale bar 50 µm. *P < 0.05, **P < 0.01, ***P < 0.001 *vs* non-treated cells in each siR group; ^#^*P* < 0.05, ^##^*P* < 0.01, ^###^*P* < 0.001 *vs* siR Control; ^σ^*P* < 0.05^, σσ^*P* < 0.01, ^σσσ^*P* < 0.001 Lvt+Baf *vs* Lvt treatment.

Autophagy has shown to be a key process in the modulating actions of lenvatinib on NRP1 expression. Considering the double-edged role of autophagy in cancer [16] and the interesting role of NRP1 in the lenvatinib-derived inhibition of HCC cell proliferation and migration, we decided to assess the relationship between them (Table 1, Fig. 5). Firstly, we evaluated the potential correlation between the receptor NRP1 and autophagy-related genes in human HCC samples (Table 1). We obtained that transcriptional expression of NRP1 is positively correlated with up to 59 genes related to autophagy, ranging from the lowest Pearson-CC of +0.31 to the strongest Pearson-CC of +0.60 (Table 1). Moreover, statistical significance (*P* < 0.0001) was reached for all these correlations observed.

**Table 1:** Significantly correlated genes from autophagy with NRP1 in human HCC samples

Abbreviation	Full gene name	Pearson-CC
BCL2L1	BCL2 like 1	+0.60
MTMR3	Myotubularin-related protein 3	+0.60
RRAGB	Ras related GTP binding B	+0.60
CFLAR	CASP8 and FADD like apoptosis regulator	+0.58
AKT3	AKT serine/threonine kinase 3	+0.57
BCL2	BCL2 apoptosis regulator	+0.56
SH3GLB1	SH3 domain containing GRB2 like, endophilin B1	+0.55
MAPK3	Mitogen-activated protein kinase 3	+0.54
GABARAPL2	GABA type A receptor-associated protein like 2	+0.53
ATG16L1	Autophagy-related 16 like 1	+0.52
MAP2K1	Mitogen-activated protein kinase kinase 1	+0.51
RRAGA	Ras related GTP binding A	+0.51
TRAF6	TNF receptor associated factor 6	+0.51
WDR41	WD repeat domain 41	+0.51
ATG3	Autophagy-related 3	+0.50
IGBP1	Immunoglobulin binding protein 1	+0.50
MAP1LC3B	Microtubule associated protein 1 light chain 3 beta	+0.50
MAPK1	Mitogen-activated protein kinase 1	+0.50
PPP2CB	Protein phosphatase 2 catalytic subunit beta	+0.50
RAB7A	RAB7A, member RAS oncogene family	+0.49
SMCR8	SMCR8-C9orf72 complex subunit	+0.48
RAB1A	RAB1A, member RAS oncogene family	+0.47
RRAS	RAS related	+0.47
PIK3C3	Phosphatidylinositol 3-kinase catalytic subunit type 3	+0.46
TANK	TRAF family member associated NFκB activator	+0.46
UVRAG	UV radiation resistance associated	+0.45
TSC1	TSC complex subunit 1	+0.44
DAPK1	Death associated protein kinase 1	+0.42
EIF2AK3	Eukaryotic translation initiation factor 2 alpha kinase 3	+0.42
RRAGC	Ras related GTP binding C	+0.42
MAP1LC3B2	Microtubule associated protein 1 light chain 3 beta 2	+0.41
BECN1	Beclin 1	+0.40
DAPK3	Death associated protein kinase 3	+0.40
EIF2S1	Eukaryotic translation initiation factor 2 subunit alpha	+0.40
PIK3R4	Phosphatidylinositol 3-kinase regulatory subunit 4	+0.40
PDPK1	3-phosphoinositide dependent protein kinase 1	+0.39
PPP2CA	Protein phosphatase 2 catalytic subunit Alpha	+0.39
TBK1	TANK binding kinase 1	+0.39
HMGB1	High mobility group box 1	+0.38
ATG4B	Autophagy-related 4B	+0.36
ATG4C	Autophagy-related 4C	+0.36
ATG7	Autophagy-related 7	+0.36
PRKACB	Protein kinase cAMP-activated catalytic subunit beta	+0.36
ATG9A	Autophagy-related 9A	+0.35
RRAS2	RAS related 2	+0.35
ITPR1	Inositol 1,4,5-trisphosphate receptor type 1	+0.34
RHEB	Ras homolog, mTORC1 binding	+0.34
NRAS	NRAS proto-oncogene, GTPase	+0.33
STX17	Syntaxin 17	+0.33
ATG2B	Autophagy-related 2B	+0.32
KRAS	KRAS proto-oncogene, GTPase	+0.32
MAP3K7	Mitogen-activated protein kinase kinase kinase 7	+0.32
PRKCD	Protein kinase C delta	+0.32
ULK2	Unc-51 like autophagy activating kinase 2	+0.32
AKT1	AKT serine/threonine kinase 1	+0.31
ATG5	Autophagy-related 5	+0.31
GABARAP	GABA type A receptor-associated protein	+0.31
GABARAPL1	GABA type A receptor-associated protein like 1	+0.31
ZFYVE1	Zinc finger FYVE-type containing 1	+0.31

*Pearson-CC* Pearson-coefficient correlation.

Based on these findings, we further analyzed the relationship between NRP1, autophagy and lenvatinib efficacy in our in vitro models of human HCC. As previously observed, combination of bafilomycin A1 with lenvatinib partially restored NRP1 protein levels; however, when NRP1 was silenced autophagy blockade did not prevent NRP1 downregulation (Fig. 5a, b, Supplementary Fig. S5a). Cell proliferation and migration processes were also assessed in these conditions, exhibiting a synergistic inhibition effect on cell viability (Fig. 5c), colony formation ability (Fig. 5d) and Ki67 proliferation index (Fig. 5e, Supplementary Fig. S5b) when HCC cells were treated with lenvatinib after NRP1 silencing. Furthermore, we demonstrated that autophagy blockage partially prevented antitumor effects of lenvatinib; nevertheless, NRP1 silencing prevented this loss of in vitro effectiveness of lenvatinib even in presence of bafilomycin A1 (Fig. 5c–e).

Regarding cell migration, autophagy inhibition also reduced the lenvatinib inhibitory effects, increasing the wound closure ability of the HCC cells in the presence of the drug (Fig. 5f, Supplementary Fig. S6). Although in this analysis NRP1 silencing and lenvatinib treatment did not show a synergy, the silencing strategy impeded the efficacy inhibition exerted by bafilomycin A1 on lenvatinib, thus avoiding loss of lenvatinib activity on cell migration (Fig. 5f, Supplementary Fig. S6).

Altogether, autophagy-dependent degradation of NRP1 seems to be a crucial mechanism in the loss of lenvatinib efficacy, which could be modulated by HCC cells during drug resistance development in order to avoid antitumor actions of lenvatinib. Therefore, NRP1 could be supposed as an interesting molecular target in human HCC in order to prevent autophagy-related lenvatinib resistance.

### NRP1 was downregulated under a hypoxic microenvironment through autophagy induction in HCC cells

**Fig. 6:**
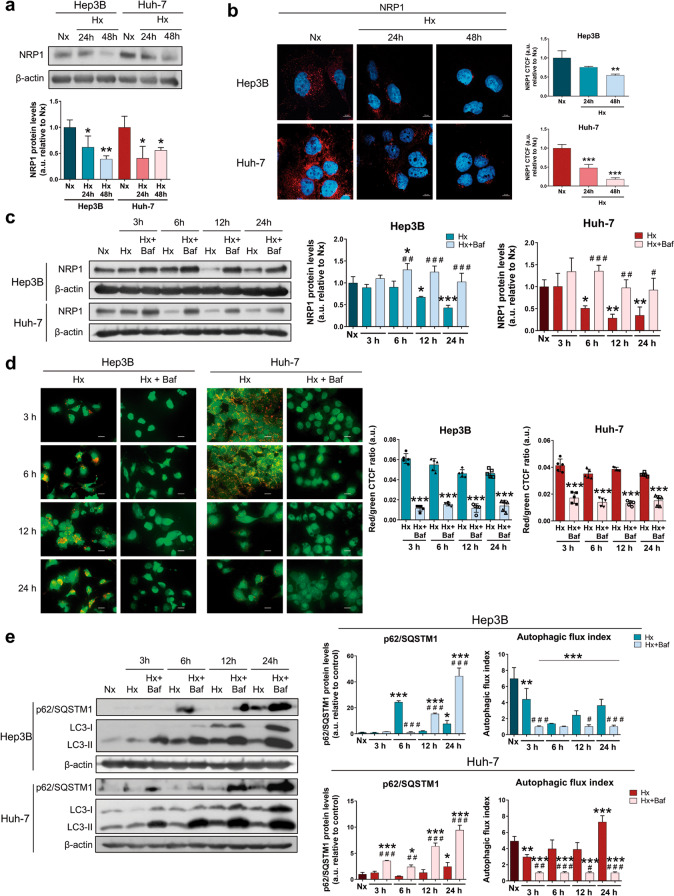
Analysis of the modulation on NRP1 protein levels by an in vitro hypoxic microenvironment. Hypoxia (Hx) was induced by incubating HCC cell lines with 100 µM CoCl_2_ for the corresponding period of times. Protein expression of NRP1 was analyzed by (**a**) Western blot and (**b**) ICC. Bar graphs from (**b**) represent the NRP1 CTCF ratio. Magnification 63× and scale bar 10 µm. *P < 0.05, **P < 0.01, ***P < 0.001 *vs* normoxia (Nx). **c** NRP1 expression was also determined by Western blot in normoxia (Nx) and after hypoxia induction and/or autophagy inhibition by treatment with 100 nM bafilomycin A1 (Baf). *P < 0.05, **P < 0.01, ***P < 0.001 *vs* Nx; ^#^*P* < 0.05, ^##^*P* < 0.01, ^###^*P* < 0.001 Hx+Baf *vs* Hx for each time point. **d** Autophagy was evaluated through analysis of autolysosome cell content by acridine orange staining and fluorescence microscopy. Magnification 40×, scale bar 25 µm. Bar graphs represent the quantification of red/green CTCF ratio (*n* = 5). ***P < 0.001 *vs* Hx for each time point. **e** Protein levels of p62/SQSTM1 and LC3 (LC3-I and LC3-II) were analyzed by Western blot. LC3 turnover assay was performed to determine the autophagic flux index. *P < 0.05, **P < 0.01, ***P < 0.001 *vs* Nx; ^#^*P* < 0.05, ^##^*P* < 0.01, ^###^*P* < 0.001 Hx+Baf *vs* Hx for each time point. Data from (**a**), (**b**), (**c**) and (**e**) are represented as mean values of arbitrary units (a.u.) ± SD (*n* = 3)^,^ showing a representative immunoblot.

Together with autophagy, the hypoxia-derived response has been also closely related to chemotherapeutic failure and drug resistance acquisition in HCC [4, 5]. For this reason, we analyzed the likely modulation derived from the induction of a hypoxic microenvironment on NRP1 expression and the role of the autophagy process through stabilization of HIFs with the employment of CoCl_2_ as hypoximimetic (Fig. 6). Results displayed a significant downregulation of NRP1 protein levels after 24 h and 48 h of hypoxia induction (Fig. 6a, b, Supplementary Fig. S7). Since autophagy showed to be the main process responsible for lenvatinib-derived downregulation of NRP1, we also tested if the same modulation was being conducted under hypoxia. Interestingly, after 12 h of hypoxia induction, a marked decrease in NRP1 expression was observed, but also a recovery by bafilomycin A1 administration not only in the selected cell lines Hep3B and Huh-7 (Fig. 6c), but this was even observed in the HepG2 cell line, which experienced a recovery of NRP1 protein levels after autophagy blockade (Supplementary Fig. S8). In the case of the Hep3B and Huh-7 lines, an efficient inhibition of hypoxia-derived autophagy was achieved by bafilomycin A1 (Fig. 6d, e). Results showed that the autophagolysosome content was significantly reduced after bafilomycin A1 administration (Fig. 6d), an accumulation of p62/SQSTM1 and LC3-II and a strong decrease of the autophagic flux index were also osberved when bafilomycin A1 was added (Fig. 6e). Therefore, NRP1 degradation by hypoxia-induced autophagy could be an interesting cell mechanism and, together with the extensive processes already known, constitute the complex response to hypoxia in HCC.

### HIF-1α modulated NRP1 expression and was involved in the loss of lenvatinib efficacy derived from the autophagy-dependent degradation of NRP1 as part of the hypoxia response

**Fig. 7:**
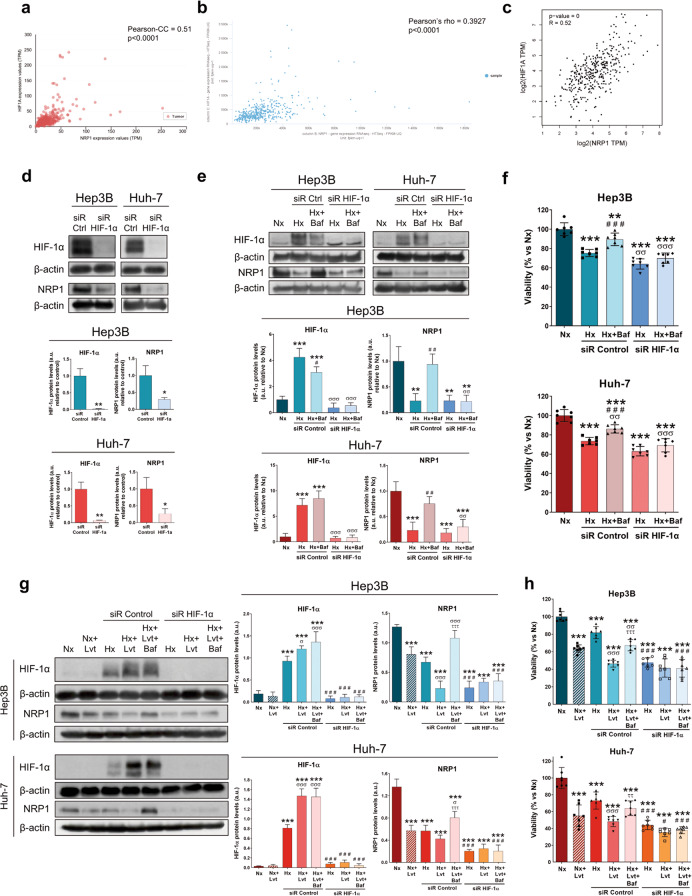
Study of the HIF-1α-dependent modulation of NRP1 expression and its role on the autophagy-derived regulation of the hypoxia response and the loss of lenvatinib efficacy. Plots of gene expression correlation between NRP1 and HIF-1α are shown, with the corresponding correlation coefficients and *p*-values obtained from the (**a**) UALCAN, (**b**) UCSC Xena and (**c**) GEPIA databases. All the following assays were performed 48 h post-silencing with the last 24 h of treatment with 100 µM CoCl_2_ to induce hypoxia (Hx), 100 nM bafilomycin A1 (Baf) and/or 2.5 µM lenvatinib (Lvt) in both HCC cell lines Hep3B and Huh-7. Protein expression was analyzed by Western blot and cell viability by MTT assay. **d** Protein levels of HIF-1α and NRP1 after HIF-1α silencing in hypoxia. *P < 0.05, **P < 0.01 *vs* siR Control. **e** Protein levels of HIF-1α and NRP1 and (**f**) cell viability after HIF-1α silencing under hypoxia and in combination with bafilomycin A1. **P < 0.01, ***P < 0.001 *vs* Nx; ^##^*P* < 0.01, ^###^*P* < 0.001 *vs* Hx; ^σσ^*P* < 0.01, ^σσσ^*P* < 0.001 *vs* siR Control. **g** Protein levels of HIF-1α and NRP1 and (**h**) cell viability after HIF-1α silencing under hypoxia and in combination with lenvatinib and/or bafilomycin A1. ***P < 0.001 *vs* Nx; ^#^*P* < 0.05, ^###^*P* < 0.001 *vs* siR Control; ^σ^*P* < 0.05, ^σσ^*P* < 0.01, ^σσσ^*P* < 0.001 Hx+Lvt *vs* Hx; ^ττ^*P* < 0.01, ^τττ^*P* < 0.001 Hx+Lvt+Baf *vs* Hx+Lvt. Data from (**d**), (**e**) and (**g**) are represented as mean values of arbitrary units (a.u.) ± SD (*n* = 3), showing one representative immunoblot. Data from (**f**) and (**h**) are represented as % of mean values relative to normoxia (Nx) ± SD (*n* = 7).

The hypoxic microenvironment has shown to be a relevant mechanism on cancer progression and is mainly modulated by the HIF-1α, which has been closely related to development of drug resistance in HCC [5, 6]. Based on the results obtained and considering the interesting role of HIF-1α-associated response to hypoxia on loss of therapeutic efficacy, we evaluated the correlation between NRP1 and HIF-1α, as well as the modulation on the lenvatinib effects on both HCC lines (Fig. 7). At first, we determined the potential gene correlation of NRP1 with HIF-1α in human HCC samples by a comprehensive analysis in three different databases. We obtained a strong positive correlation between both genes, finding the correlation coefficients of +0.51 (Fig. 7a), +0.39 (Fig. 7b) and +0.52 (Fig. 7c), with statistical significance in all cases (*P* < 0.0001). We further performed an in vitro analysis by specifically silencing HIF-1α. Interestingly, we observed that an effective HIF-1α silencing under hypoxia led to a significant downregulation of NRP1 protein levels (Fig. 7d). Additionally, when HIF-1α silencing was combined with bafilomycin A1, HIF-1α was slightly decreased only in Hep3B and successfully silenced in both cell lines (Fig. 7e). On the other hand, NRP1 expression decreased under hypoxia and, as expected, was recovered when autophagy was inhibited; while HIF-1α gene silencing prevented this increase on NRP1 expression derived from bafilomycin A1 (Fig. 7e). We also found that acute hypoxia induction decreased cell viability of both HCC lines, showing a synergy when HIF-1α was silenced. Autophagy blockade increased Hep3B and Huh-7 viability; however, HIF-1α silencing prevented this reversal effect of bafilomycin A1, thus improving the inhibition exerted by hypoxia and HIF-1α silencing (Fig. 7f).

Since autophagy inhibition could act as a relevant mechanism in the loss of lenvatinib efficacy through NRP1 modulation, as well as in the HIF-1α-associated response to hypoxia, crucial in the adaptive cellular response to chemotherapy, we decided to elucidate the interesting relationship among them in both HCC cell lines. Lenvatinib treatment under hypoxia increased HIF-1α protein expression, while augmented the NRP1 downregulation exerted by hypoxia (Fig. 7g). Remarkably, autophagy blockade by bafilomycin A1 restored NRP1 protein levels even in presence of lenvatinib and a hypoxic microenvironment, while no changes were observed in HIF-1α expression (Fig. 7g). When HIF-1α was specifically silenced, these alterations were not observed in both HIF-1α and NRP1 expression, preventing the upregulation of NRP1 caused by autophagy inhibition (Fig. 7g). Regarding cell viability, similar findings were obtained in which the synergistic effects of lenvatinib and hypoxia induction on decreasing cell viability were partially restored by bafilomycin A1. Likewise, HIF-1α silencing not only increased the inhibitory actions of combined lenvatinib and hypoxia, but also prevented the loss of effectiveness derived from autophagy blockade (Fig. 7h).

Altogether, these results suggest that NRP1 is directly modulated by HIF-1α under hypoxia and that autophagy plays a crucial role in lenvatinib efficacy and cell response to hypoxia through NRP1 modulation. Therefore, not only NRP1, but also HIF-1α, could act as potential targets in order to prevent therapeutic failure overpassing an adaptive cellular response through autophagy modulation.

## Discussion

**Fig. 8:**
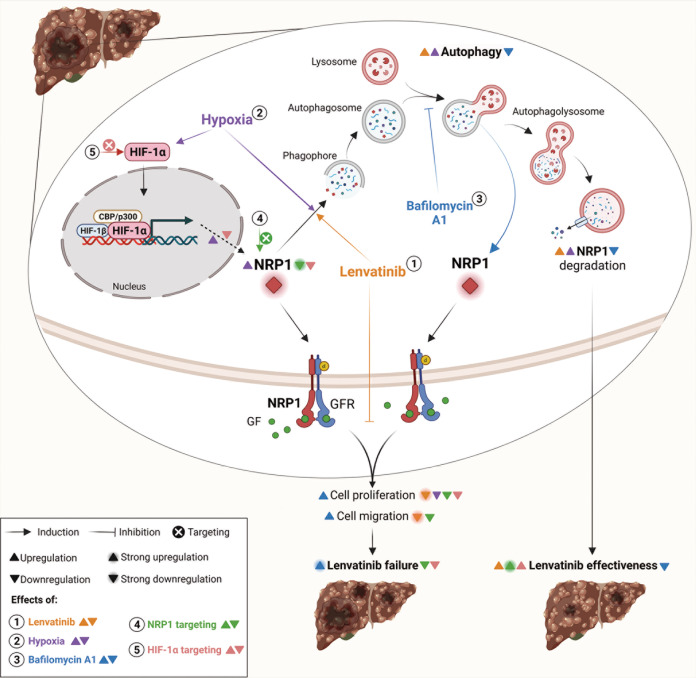
Schematic illustration of the mechanisms here elucidated as responsible for the loss of lenvatinib efficacy in human HCC. Through autophagy, (**1**) lenvatinib promotes NRP1 degradation and strongly decreases cell proliferation and migration. (**2**) Hypoxic conditions act by inducing autophagy and, therefore, NRP1 degradation, but also the higher expression of HIF-1α raised NRP1 protein levels. (**3**) When autophagy is inhibited by bafilomycin A1, NRP1 levels increase and the antitumor actions of lenvatinib experience a reduction, increasing proliferation and migration abilities of HCC cells. Targeting not only (**4**) NRP1, but also (**5**) HIF-1α, enhances antitumor effects of lenvatinib and could prevent therapeutic failure derived from autophagy inhibition by HCC cells. This figure was created with BioRender.com.

HCC remains a global health problem [2] and, despite great efforts to generate effective targeted drugs, human HCC cells manage to develop molecular strategies that lead to therapeutic failure [3, 4]. The high heterogeneity that characterizes human HCC is also in line with the broad variety of cellular processes involved in chemoresistance acquisition [3, 4, 10], in which the double-edged process of autophagy and the hypoxia-derived response stand as crucial mechanisms [4–6]. Due to its ability to interact with numerous growth factor receptors, such as VEGF/VEGFR, NRP1 has shown to modulate several signaling pathways involved in tumor progression [7, 9]. Consequently, this co-receptor has recently arisen as an interesting protein with potential implication in tumor development and drug responsiveness [7, 8]. This study revealed a mechanistic modulation of NRP1 through autophagy in human HCC cells strongly associated with the loss of lenvatinib effectiveness, in which HIF-1α-dependent response to hypoxia may also play a crucial role by directly modulating NRP1 expression and lenvatinib efficacy (Fig. 8).

Although NRP1 expression was firstly identified in the central nervous system, this receptor is expressed in different tissues [9], being overexpressed in several tumor types, including HCC [9, 33, 34]. Increased NRP1 expression in human HCC tissue was also observed in this study, as well as higher NRP1 levels in advanced tumor stages and nodal metastasis status. Despite several investigations performed with in vitro and in vivo HCC models have described antitumor properties of lenvatinib [35, 36], the role of NRP1 in lenvatinib efficacy has not been previously evaluated. In this line, we observed that NRP1 downregulation by lenvatinib is partially responsible for its antitumor effects on HCC cell proliferation and migration. Likewise, several proteins with different cell functions have been described to be potential targets for increasing lenvatinib effectiveness in human HCC, such as ADAMTS-like protein 5 (ADAMTSL5) [37], stomatin-like protein 2, mitochondrial (STOML2) [38] or fibroblast growth factor receptor 1 (FGFR1) [39]; but only STOML2 have shown to mediate the anti-migratory effects of lenvatinib [38], as we observed with NRP1.

Autophagy is a widely studied process with an interesting role in tumor progression and chemotherapeutic efficacy [15]. In this study, autophagy has shown to be the main process responsible for modulation of NRP1 protein levels, being placed as a key mechanism in the loss of therapeutic effectiveness of lenvatinib in our in vitro HCC model (Fig. 8). Previous research has described a context-dependent role of autophagy in drug response in HCC, either enhancing lenvatinib efficacy [40], or promoting sorafenib failure [25]. Altogether, liver tumor cells could modulate autophagy as part of a cellular response to decrease therapeutic efficacy. Our findings showed that through autophagy blockade, NRP1 levels were restored along with cell proliferation and migration ability, leading to lower antitumor effects of lenvatinib (Fig. 8). Similarly, lenvatinib augmented autophagy and promoted cell death of the Hep3B and Huh-7 cell lines, while autophagy inhibition by Atg5 or Beclin-1 gene silencing strongly decreased the efficacy of lenvatinib and entinostat combination [36]. Moreover, an in vitro model of sorafenib resistant HCC showed that sterol-regulatory element binding protein (SREBP) cleavage-activating protein (SCAP)-derived sorafenib resistance was associated with a lower autophagy activation [41]. Higher autophagy induction also enhanced sorafenib sensitivity in Huh-7 cells with acquired sorafenib resistance [42, 43], displaying a higher cell migration ability when autophagy was not induced [42]. Nonetheless, opposite results have been also described, in which osteopontin-derived autophagy promoted chemoresistance in HCC cell lines [44], and autophagy targeting enhanced the antitumor actions of sorafenib [45]. Collectively, these findings support the potential modulation that resistant HCC cells could exert through autophagy inhibition to overcome antitumor effects of TKIs, which in our model could be mediated, at least in part, by NRP1.

In order to avoid an autophagy-associated mechanism of adaptation to lenvatinib treatment, we tested the effects of targeting NRP1 when autophagy was blocked. We observed that NRP1 silencing prevented the loss of lenvatinib antitumor actions derived from autophagy inhibition (Fig. 8). Likewise, previous reports have described that NRP1 downregulation significantly reduced cell migration and cell proliferation in two in vitro models of HCC [46, 47], as well as diminished tumor volume and vasculature in an HCC murine model [47]. Moreover, two different studies with human colorectal cancer (CRC) cells showed that after adaptation to sunitinib, CRC cells switched from a VEGFR-dependent to a NRP1/cMet-dependent pathway promoting treatment evasion, and that targeting NRP1 suppressed migration activation [48, 49]. Although autophagy has been previously described as the degradation mechanism of NRP1 under hypoxia or nutrient deprivation conditions [50], no investigations have been conducted in which the interplay between the autophagy-dependent degradation of NRP1 and drug responsiveness in cancer was fully elucidated.

Overall, these results suggest that NRP1 contributes to a higher cell survival and migration-associated abilities of HCC cells, highlighting the crucial modulation exerted by the autophagy-dependent degradation of NRP1 as a potential mechanism associated with the loss of lenvatinib effectiveness.

The hypoxia microenvironment has been closely related to the development of resistance mechanisms by tumor cells, including liver cancer cells [5, 6, 51]. We further assessed the hypoxia-derived alterations on NRP1 expression and the associated mechanisms studied. Although a hypoxic microenvironment can be induced using hypoxic chambers, in this study we employed CoCl_2_ as a hypoximimetic to induce a hypoxia-derived response. CoCl_2_ is an agent that acts by inactivating the enzymes responsible for the degradation of the HIFs and, therefore, promoting the stabilization of these factors [52]. The use of CoCl_2_ has been chosen for inducing a representative hypoxic response for several years, due to the hypoxic chamber conditions are usually different depending on the cell line analyzed. Our results indicated that hypoxia induction markedly reduced protein expression of NRP1 and, as observed by lenvatinib treatment, autophagy was the main mechanism responsible (Fig. 8). In this line, an analysis conducted with human HCC patients demonstrated that peritumoral hypoxia was significantly correlated with higher peritumoral expression of NRP1 [53]. However, opposite results have been observed, describing a decrease in NRP1 expression through autophagy in hypoxia in breast and prostate carcinoma cells [50], while raised levels were observed under hypoxia in cervical cancer [54], lung adenocarcinoma [55] and oral squamous cell carcinoma [56]. On the other hand, few studies have assessed the possible effects derived from hypoxia induction on the cellular response to lenvatinib in human HCC, while further have been performed with other different TKIs. Results have been published in which hypoxia, through a HIF-dependent response, was highly involved in the acquisition of sorafenib resistance in an in vitro model of HCC [11]. Similarly, an investigation performed with both cellular and animal models found that hypoxia-derived overexpression of STOML2 led to loss of lenvatinib sensitivity in human HCC [38]. Moreover, a specific evaluation of the derived effects from hypoxia induction on lenvatinib efficacy has been assessed in the PLC/PRF/5 hepatoma cell line [57]. Although this study employed only one cell line, results showed that the IC_50_ for lenvatinib increased under hypoxia and that HCC cells exhibited a lenvatinib resistance phenotype under hypoxia conditions that could be mediated by changes observed in the extracellular matrix, mainly associated to fibronectin [57]. Finally, a recent study also reported that ubiquitin specific peptidase 2 antisense RNA 1 (USP2-AS1) overexpression under hypoxia diminished lenvatinib efficacy by increasing HIF-1α expression in liver cancer [58]. These findings and those observed in our study provide interesting results and support the crucial role of the hypoxic microenvironment on the cellular response to lenvatinib that could drive to chemotherapeutic resistance.

HIF-1α has been widely associated with resistance acquisition in several solid tumors, including HCC, through cellular adaptation to hypoxia [10–12]. For this reason, we aimed at identifying the likely correlation between NRP1 and HIF-1α in both human HCC samples and our in vitro models of HCC, as well as to evaluate the HIF-1α-derived modulation on NRP1 expression and the associated effects on lenvatinib efficacy under hypoxia. In our study, NRP1 was strongly correlated with HIF-1α and this transcription factor directly modulated NRP1 expression (Fig. 8). Despite the key role of NRP1 in modulating angiogenesis and migration-derived processes, as well as in the development of drug resistance [7, 8], studies that assess its association with the HIF-1α-derived response to hypoxia in HCC have not been conducted. In other tumor types some interesting findings have been published, where HIF-1α interacted with the NRP1 promoter in lung adenocarcinoma and induced cell metastasis and vasculogenic mimicry [55]. Likewise, HIF-1α also modulated epithelial-to-mesenchymal transition (EMT) through the VEGFA/NRP1 axis in low Gleason grade cancers [59].

Evidences of the autophagy and hypoxia interplay have placed them as key mechanisms in therapeutic failure in HCC [14, 17]. Therefore, we analyzed the HIF-1α modulation on the role of the autophagy-dependent NRP1 degradation in lenvatinib efficacy. HIF-1α downregulation by gene silencing diminished NRP1 expression as well as cell viability, even avoiding the recovery of both NRP1 and cell viability when autophagy was blocked. Furthermore, although autophagy inhibition increased cell viability even under hypoxia and lenvatinib treatment, HIF-1α silencing significantly reduced NRP1 expression and countered this loss of lenvatinib efficacy and a possible hypoxia-mediated adaptive response of the HCC cells (Fig. 8). Similarly, autophagy blockade by beta-2 adrenergic receptor (ADRB2) in an in vivo HCC model promoted DEN-induced hepatocarcinogenesis and sorafenib resistance acquisition by HIF-1α stabilization [60]. Meanwhile, a direct association of elevated HIF-1α with decreased Beclin-1 in the promotion of HCC differentiation and progression was observed in human HCC samples [13]. According to our hypothesis, HCC cells have shown an autophagy-associated shift derived from sustained treatment with sorafenib [61] and this process was also the mechanism responsible for HCC cell adaptation to the hypoxic microenvironment [62]. Contrariwise, hypoxia induced N6-methyladenosine (METTL3) depletion and sorafenib resistance by inducing autophagy in both in vitro and in vivo models of human HCC [63]. Collectively, these results suggest that not only NRP1, but also HIF-1α, could be potential targets to hinder an autophagy-associated response by HCC cells as part of a hypoxia-derived adaptation that promote the loss of lenvatinib effectiveness.

This study provides novel findings on the molecular mechanisms associated with NRP1-dependent survival and migration of human HCC cells underlying the loss of lenvatinib effectiveness. Overall, these results displayed the key role of NRP1 in antitumor actions of lenvatinib in human HCC, where autophagy acts as a crucial mechanism involved in cell adaptation to lenvatinib treatment through NRP1 modulation, thus promoting cell resistance development, in which HIF-1α-associated hypoxia response seems to be a potential mediator. Therefore, NRP1 stands as a valuable target in advanced HCC to prevent therapeutic failure through autophagy-related lenvatinib resistance.

## Supplementary information

Supplementary Fig. S1

Supplementary Fig. S2

Supplementary Fig. S3

Supplementary Fig. S4

Supplementary Fig. S5

Supplementary Fig. S6

Supplementary Fig. S7

Supplementary Fig. S8

Supplementary Fig. S9

Supplementary Fig. S10

Supplementary Fig. S11

Supplementary Fig. S12

Supplementary Fig. S13

Supplementary Fig. S14

Supplementary Fig. S15

Supplementary Fig. S16

Supplementary Fig. S17

Supplementary Fig. S18

Supplementary Fig. S19

Supplementary figure legends
