# Supplementary figures and images for: Hepatocellular carcinoma cells loss lenvatinib efficacy in vitro through autophagy and hypoxia response-derived neuropilin-1 degradation
Source: Acta Pharmacol Sin. 2022 Nov 14;44(5):1066–82. doi: 10.1038/s41401-022-01021-2 (PMC10104874; doi:10.1038/s41401-022-01021-2)

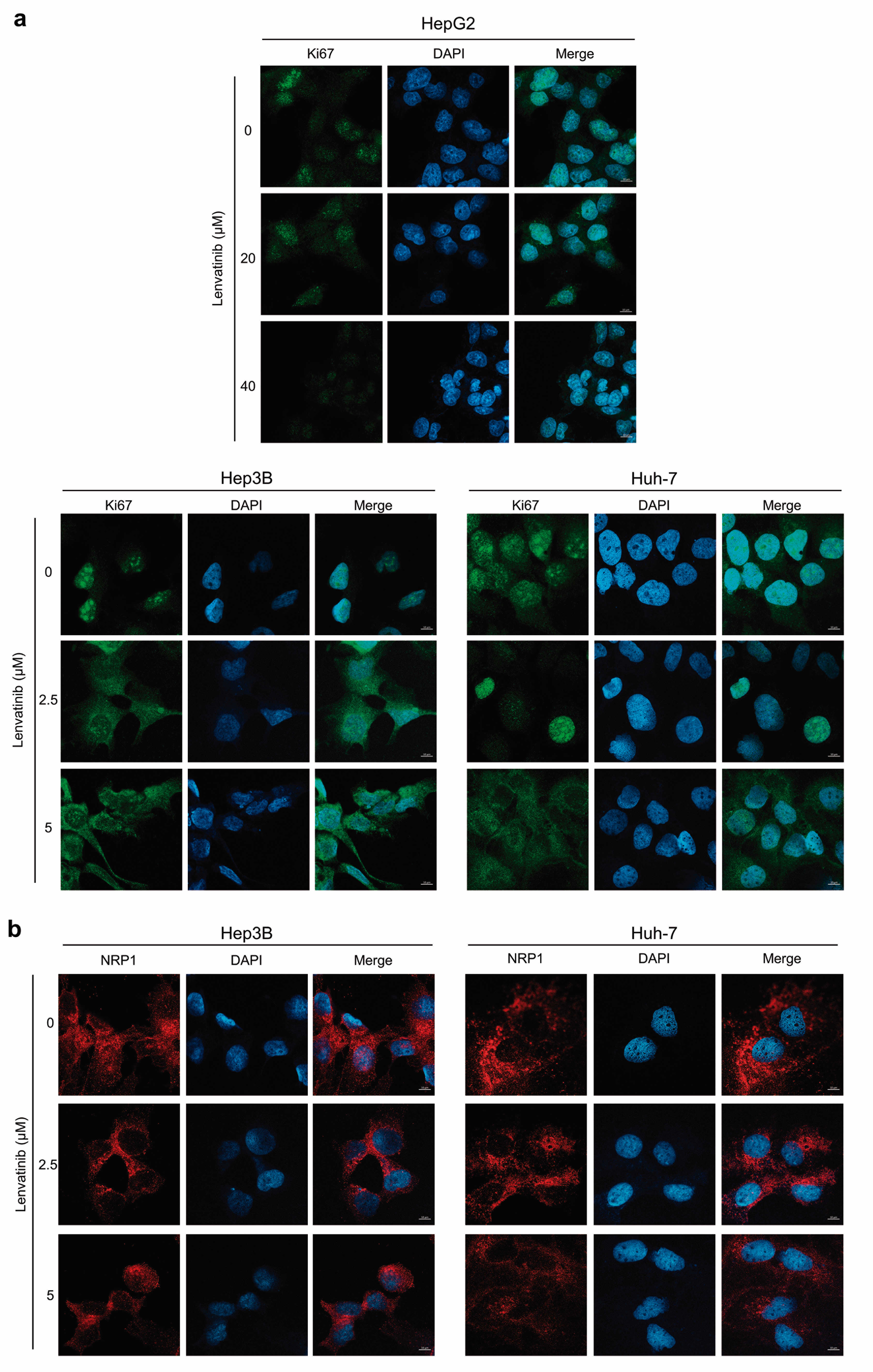

Supplement: Supplementary file 1 — Supplementary Fig. S1 [file 41401_2022_1021_MOESM1_ESM.jpg]

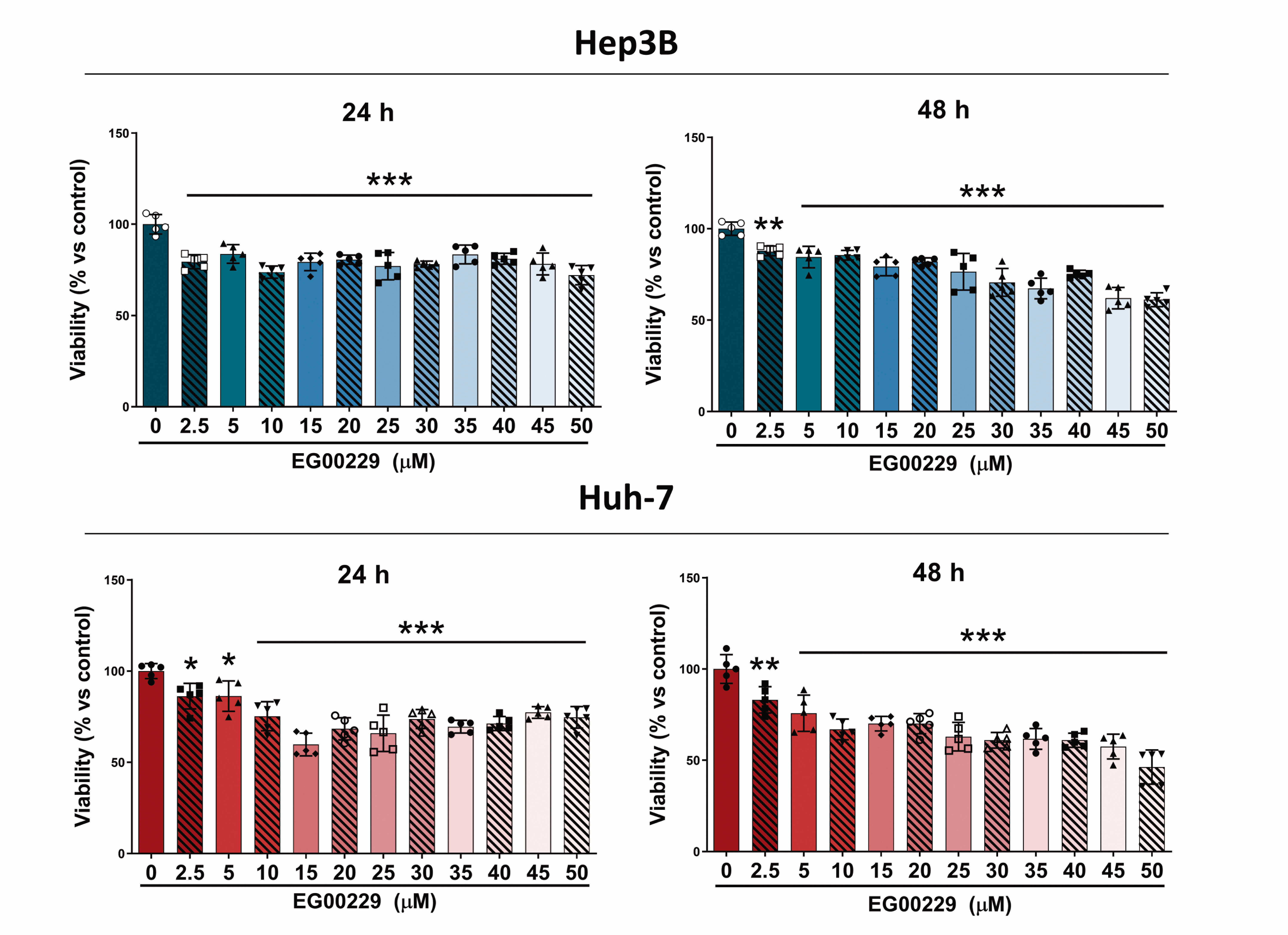

Supplement: Supplementary file 2 — Supplementary Fig. S2 [file 41401_2022_1021_MOESM2_ESM.jpg]

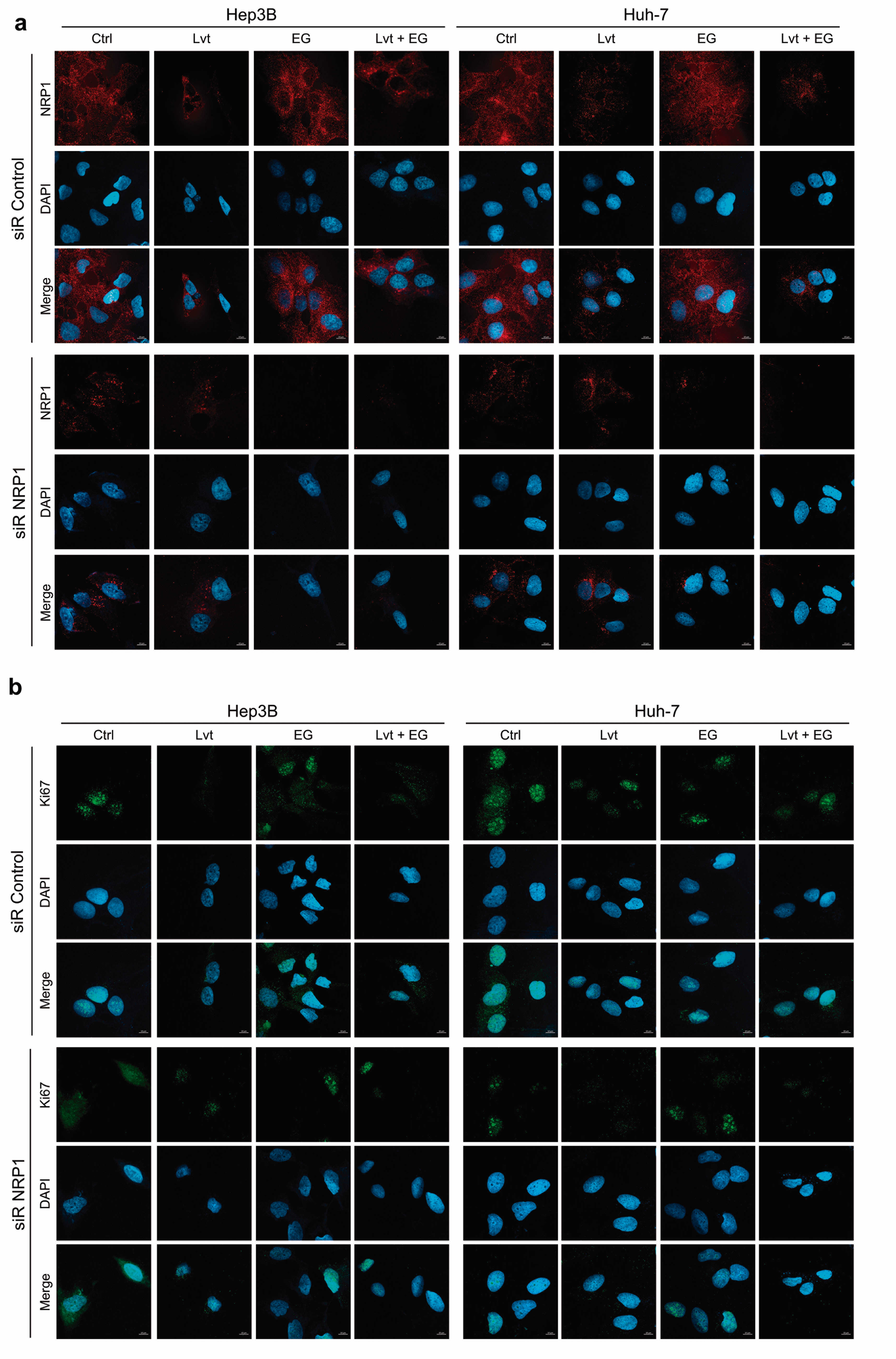

Supplement: Supplementary file 3 — Supplementary Fig. S3 [file 41401_2022_1021_MOESM3_ESM.jpg]

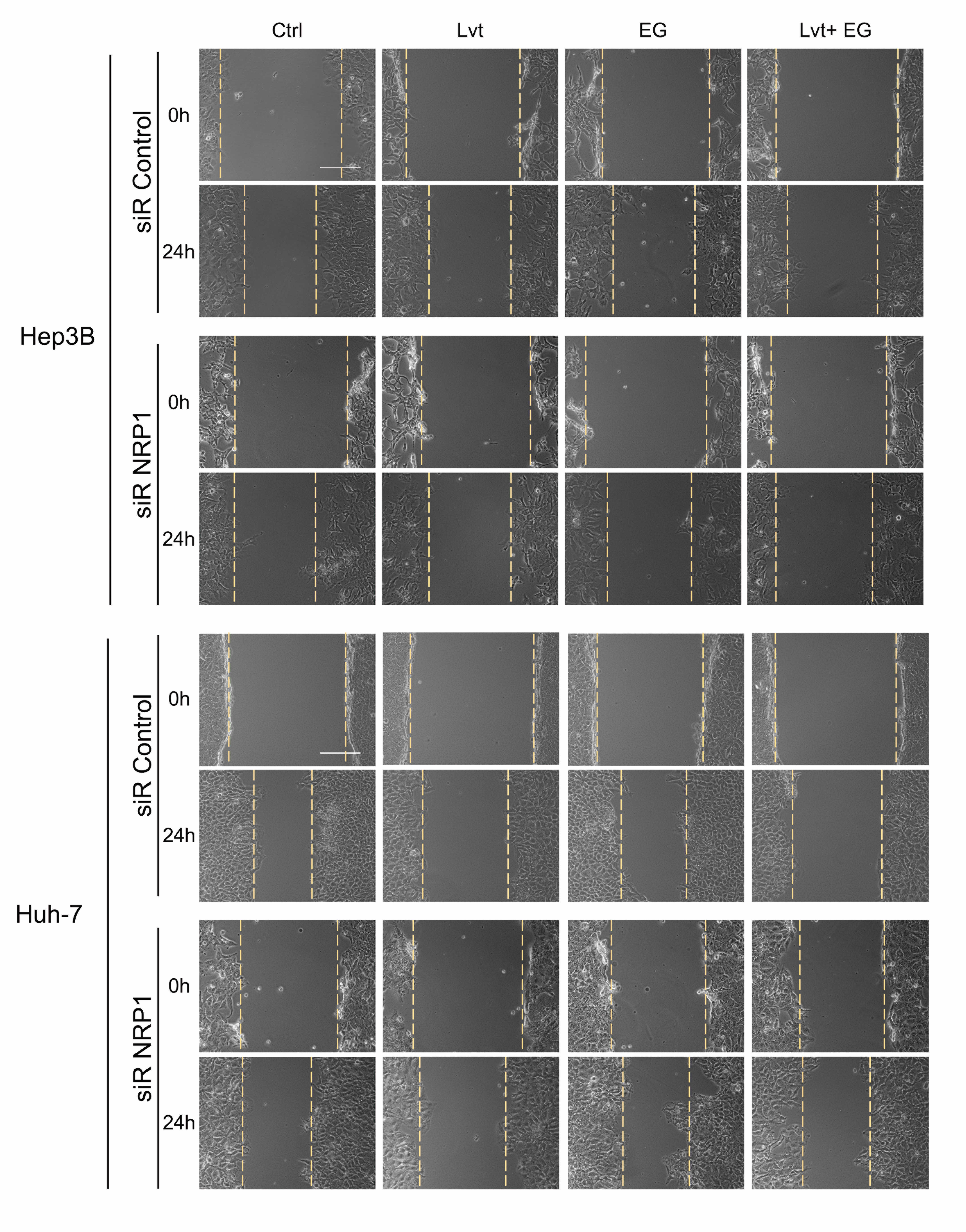

Supplement: Supplementary file 4 — Supplementary Fig. S4 [file 41401_2022_1021_MOESM4_ESM.jpg]

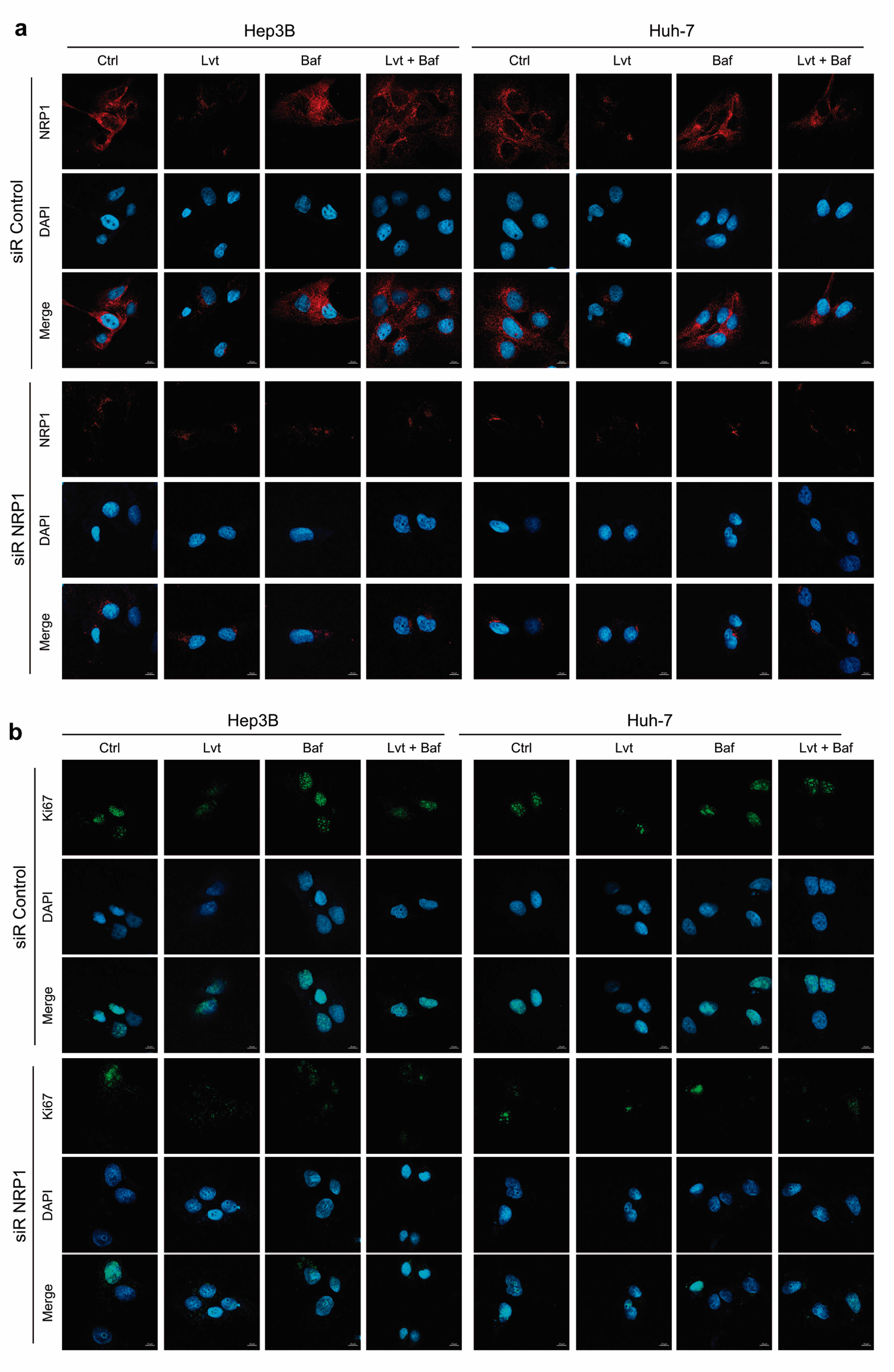

Supplement: Supplementary file 5 — Supplementary Fig. S5 [file 41401_2022_1021_MOESM5_ESM.jpg]

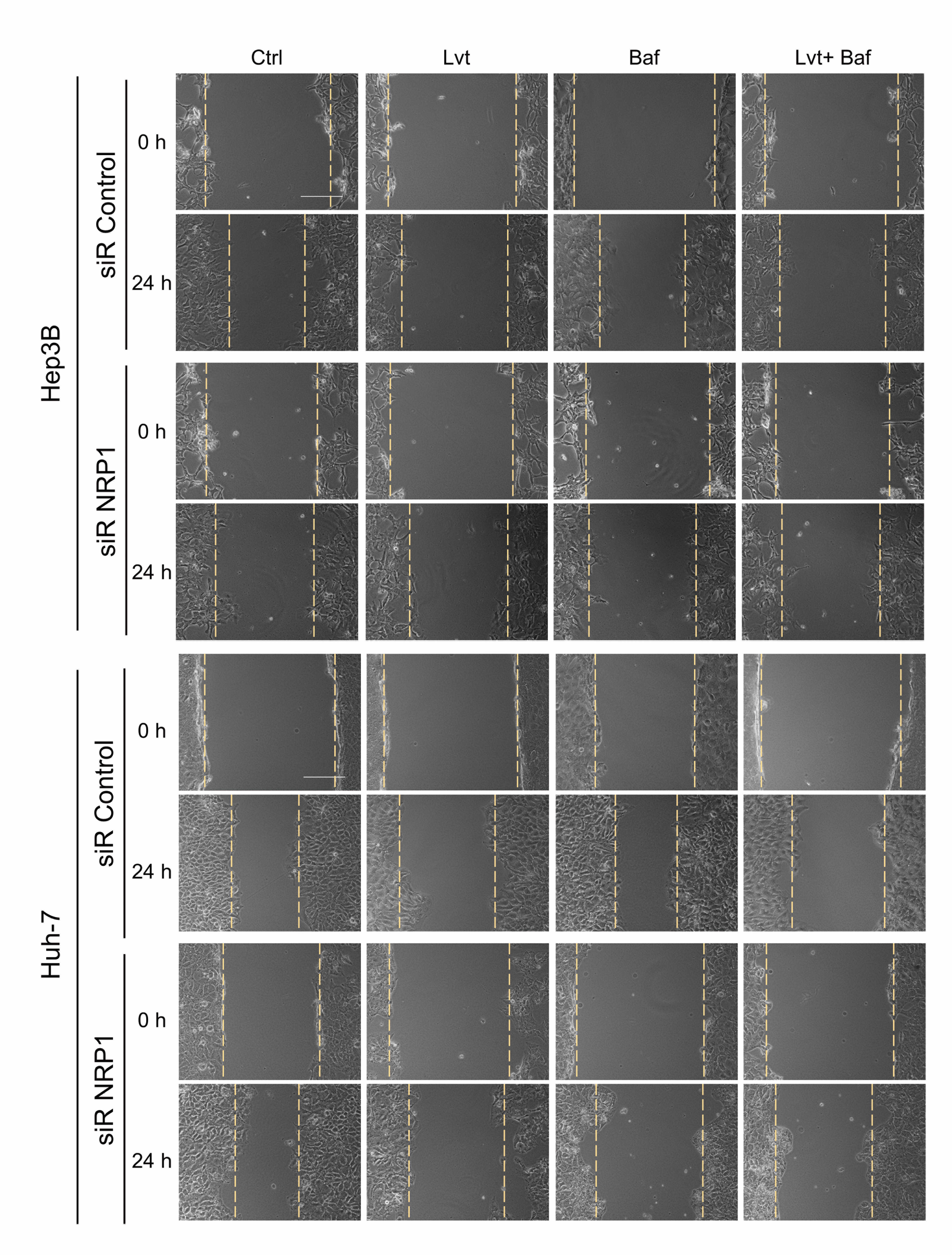

Supplement: Supplementary file 6 — Supplementary Fig. S6 [file 41401_2022_1021_MOESM6_ESM.jpg]

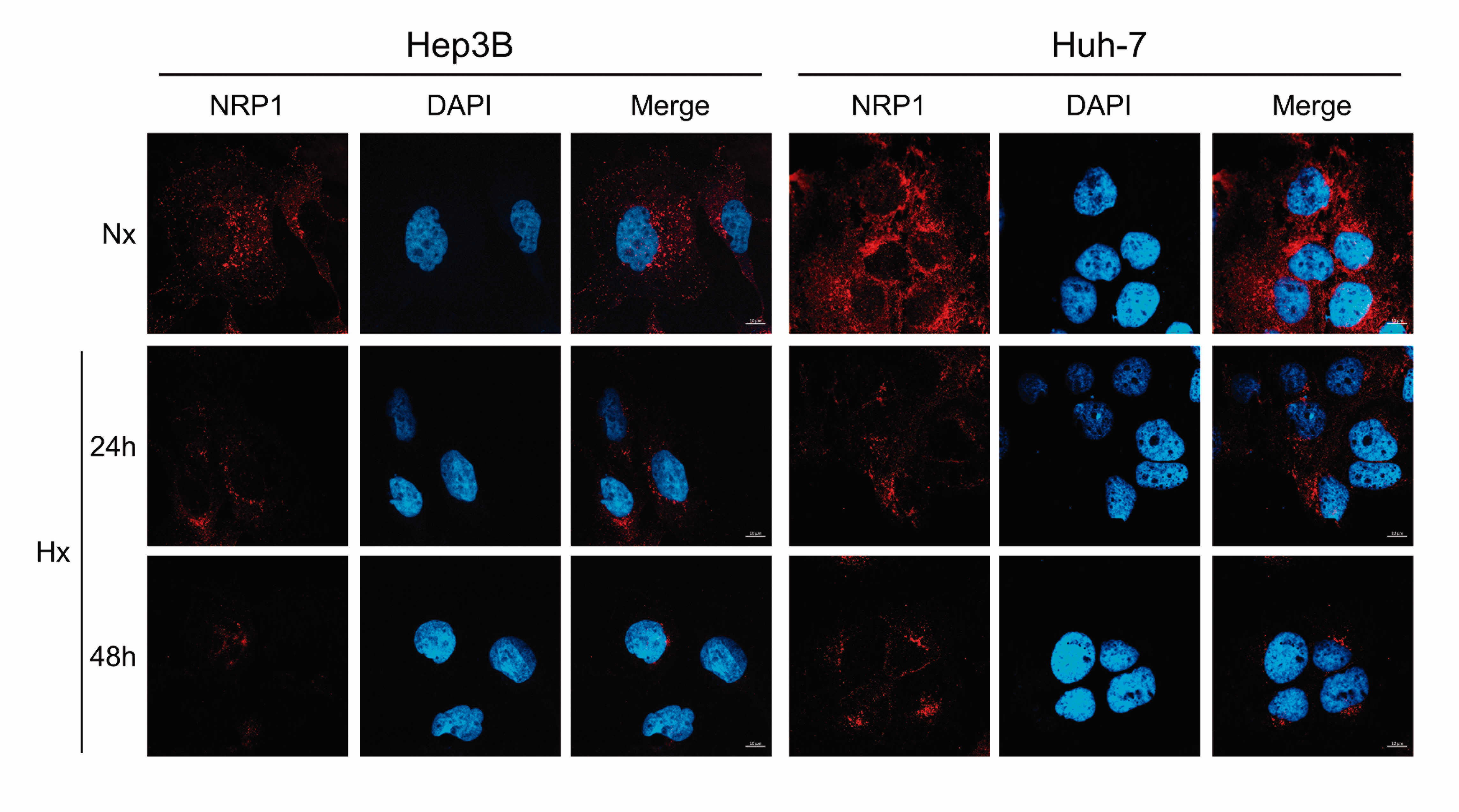

Supplement: Supplementary file 7 — Supplementary Fig. S7 [file 41401_2022_1021_MOESM7_ESM.jpg]

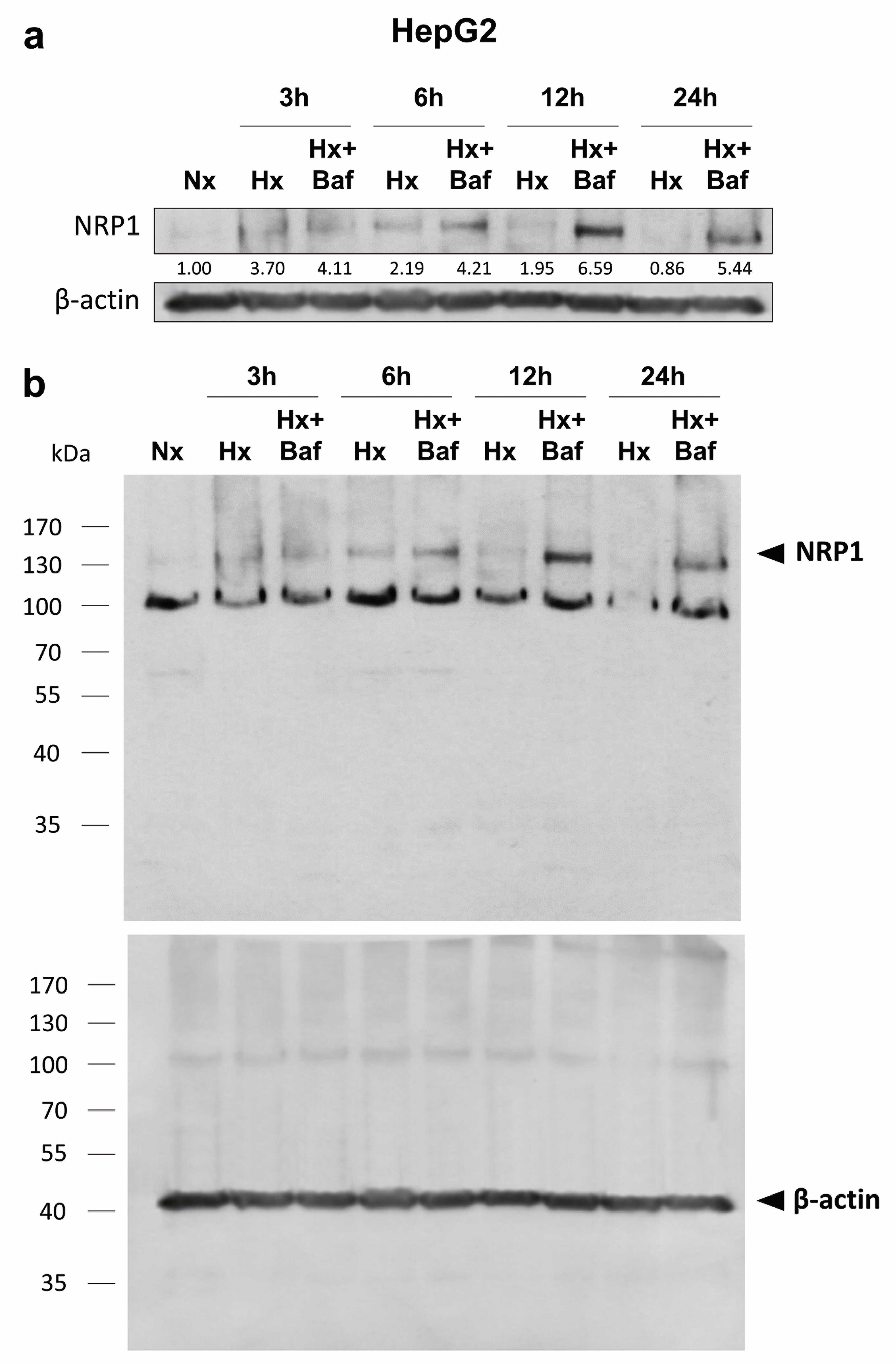

Supplement: Supplementary file 8 — Supplementary Fig. S8 [file 41401_2022_1021_MOESM8_ESM.jpg]

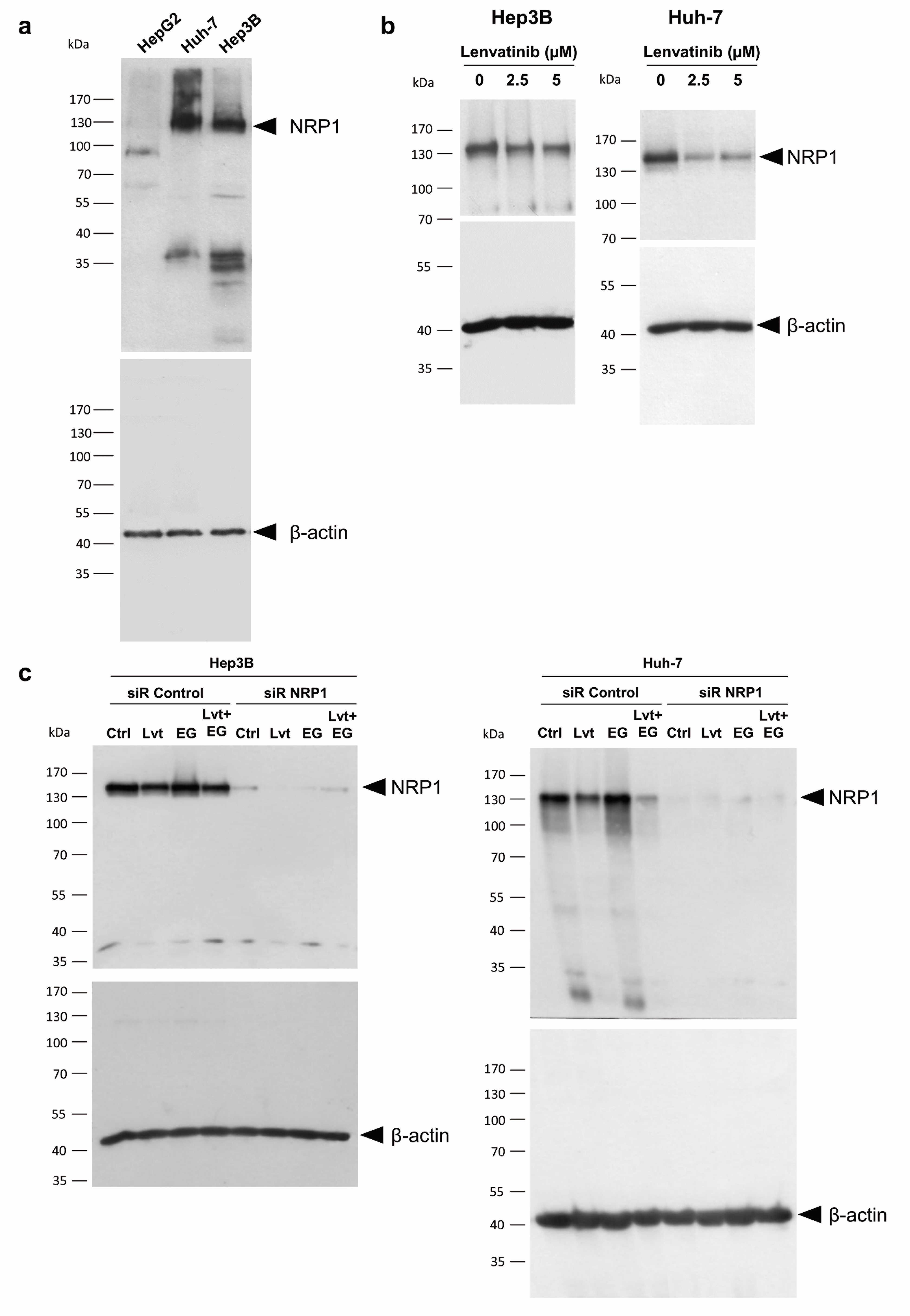

Supplement: Supplementary file 9 — Supplementary Fig. S9 [file 41401_2022_1021_MOESM9_ESM.jpg]

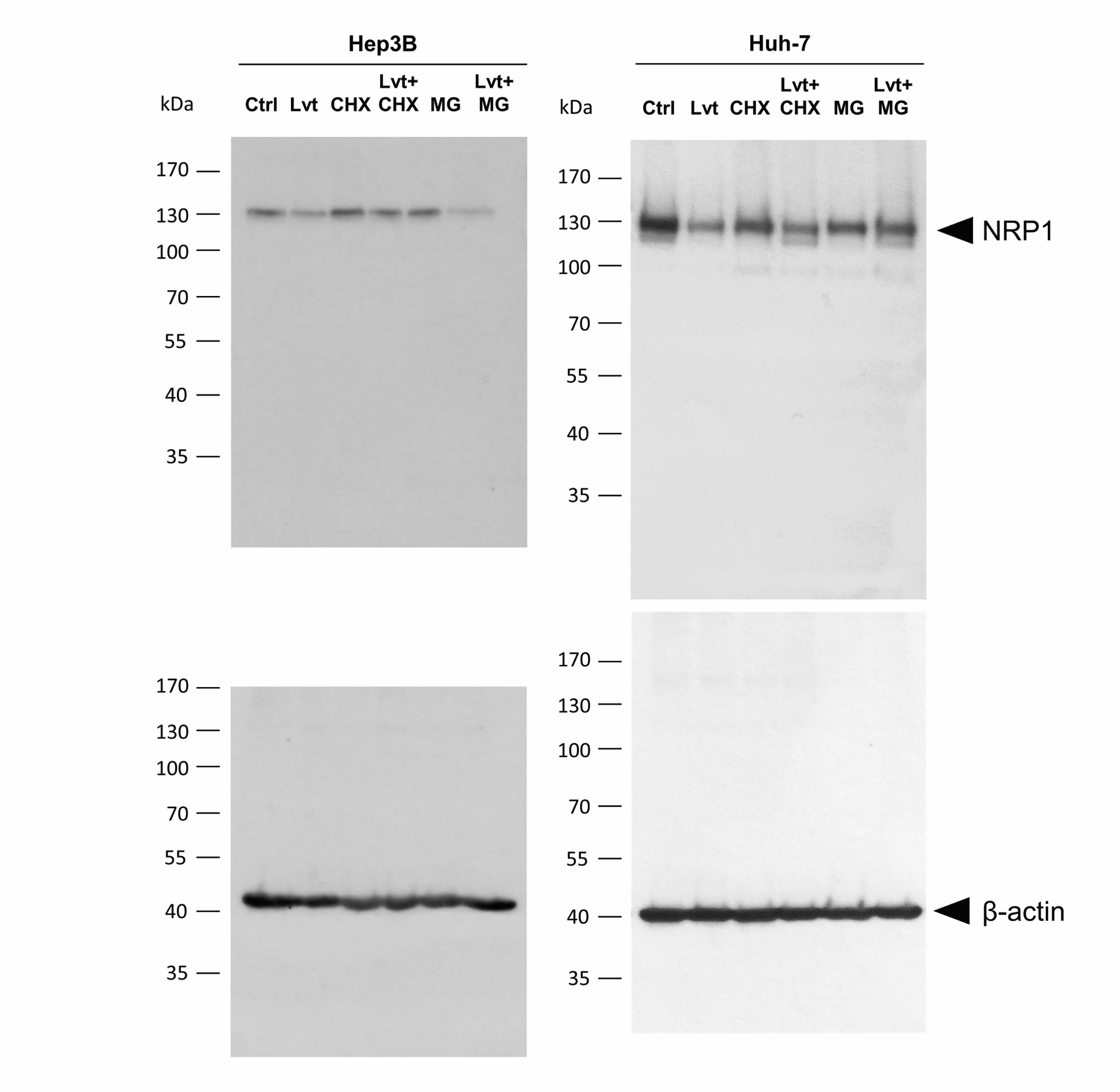

Supplement: Supplementary file 10 — Supplementary Fig. S10 [file 41401_2022_1021_MOESM10_ESM.jpg]

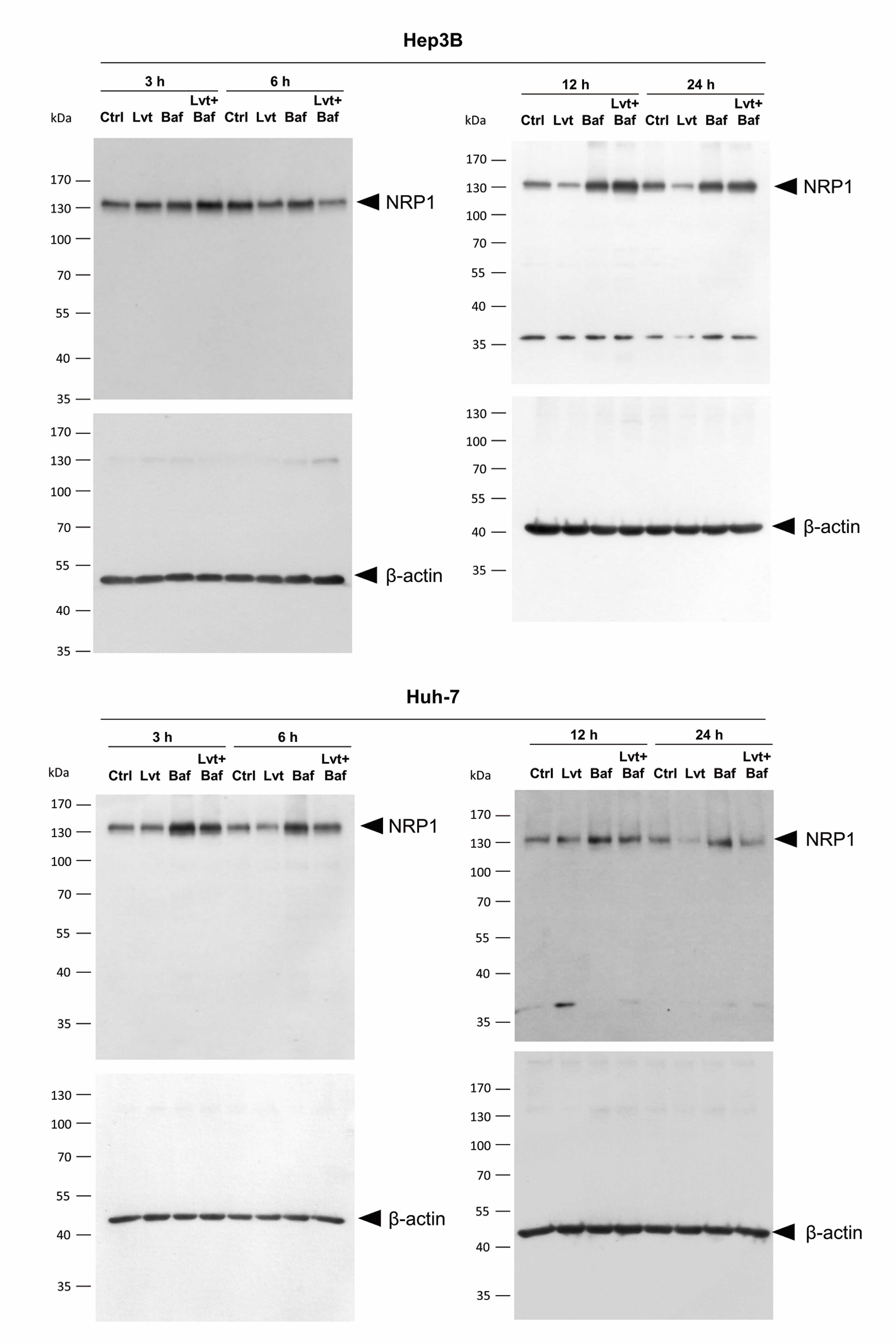

Supplement: Supplementary file 11 — Supplementary Fig. S11 [file 41401_2022_1021_MOESM11_ESM.jpg]

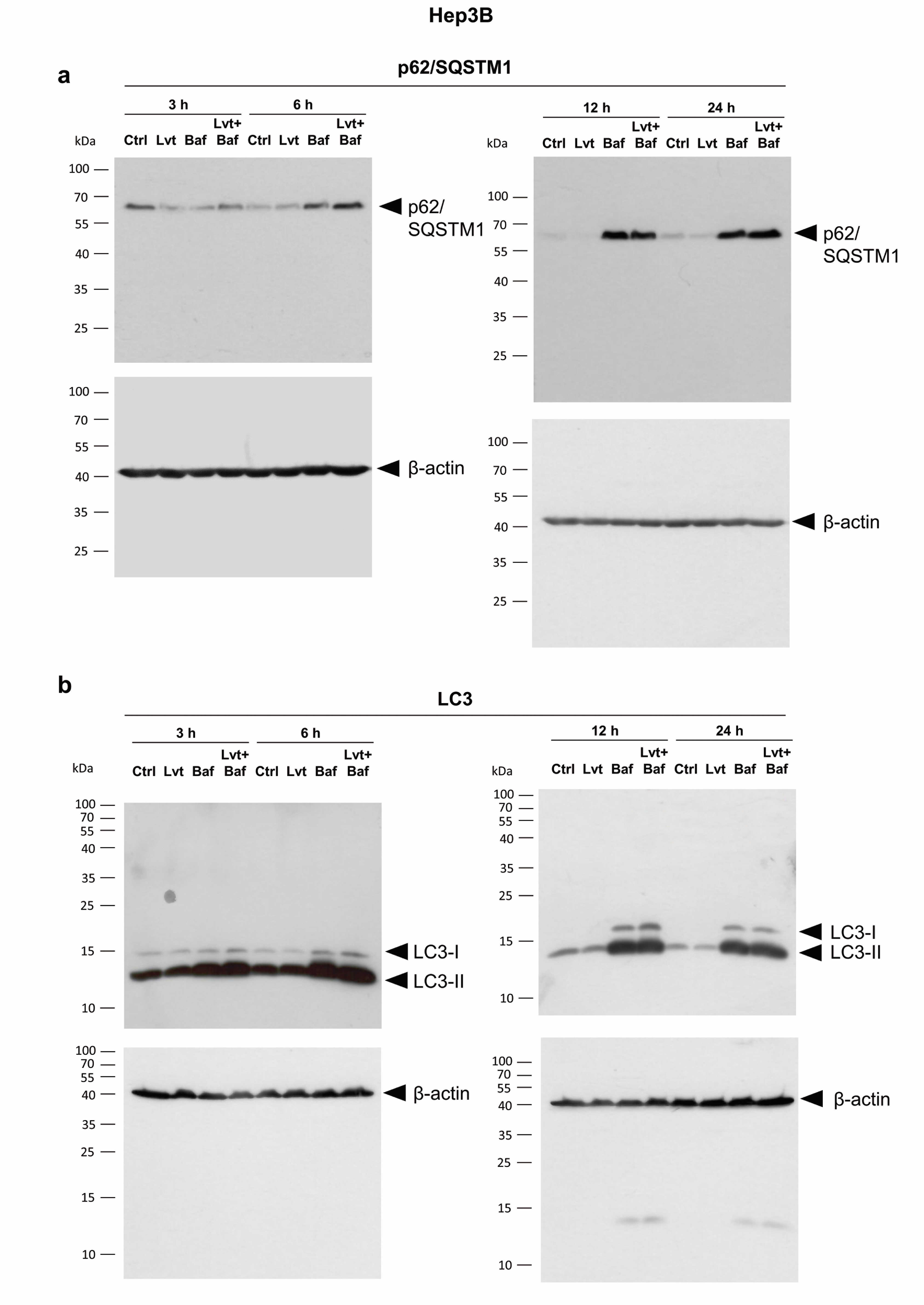

Supplement: Supplementary file 12 — Supplementary Fig. S12 [file 41401_2022_1021_MOESM12_ESM.jpg]

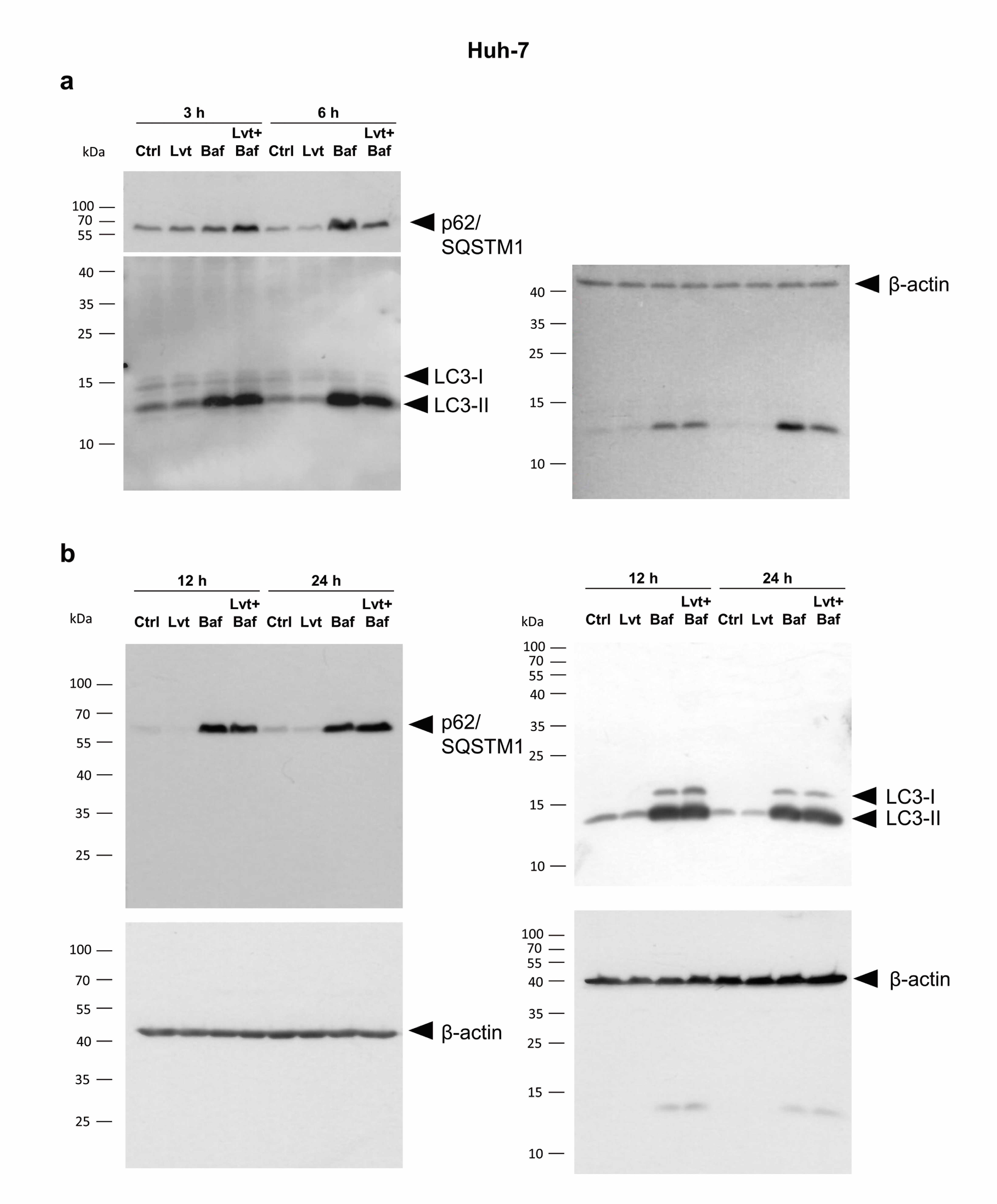

Supplement: Supplementary file 13 — Supplementary Fig. S13 [file 41401_2022_1021_MOESM13_ESM.jpg]

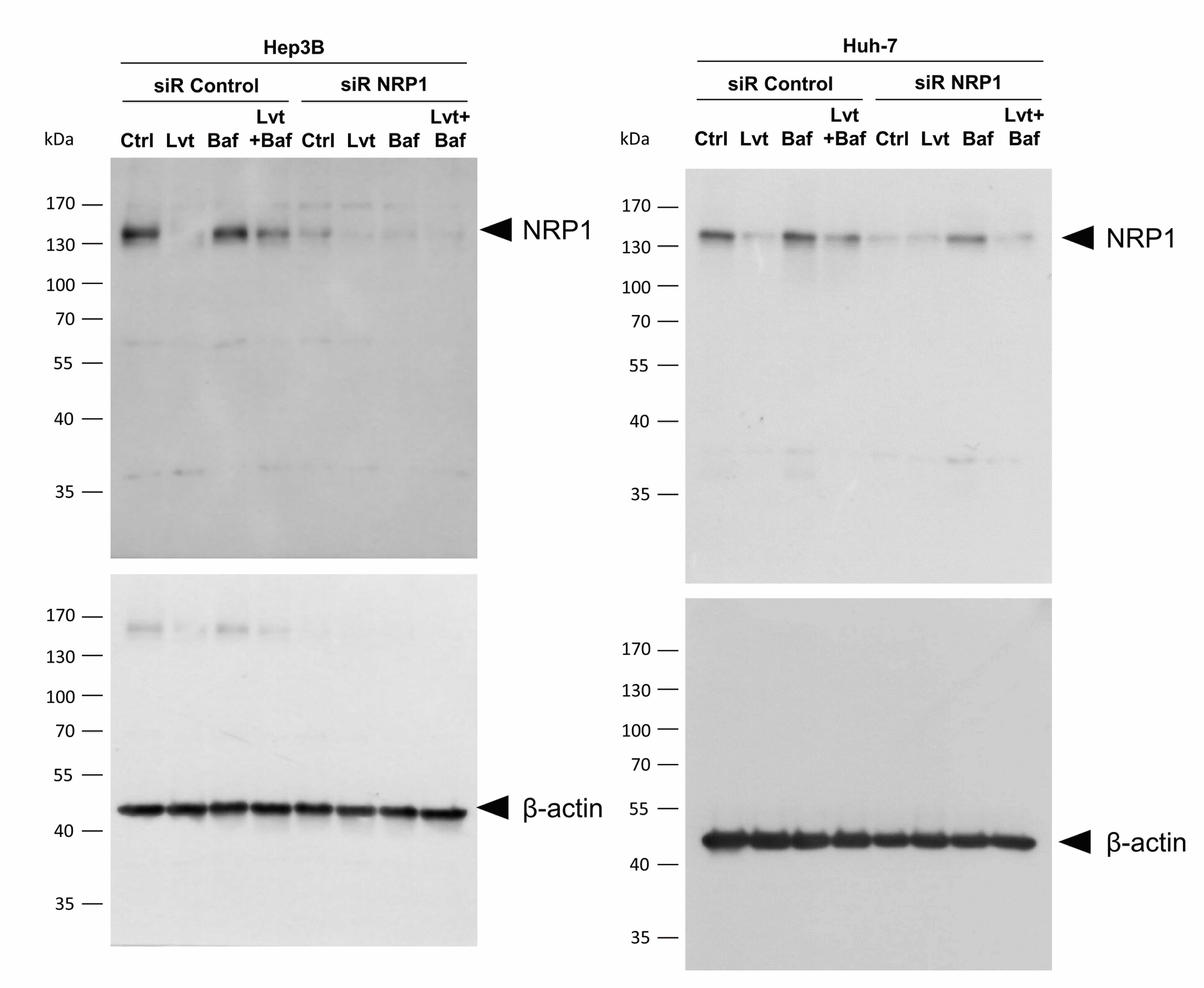

Supplement: Supplementary file 14 — Supplementary Fig. S14 [file 41401_2022_1021_MOESM14_ESM.jpg]

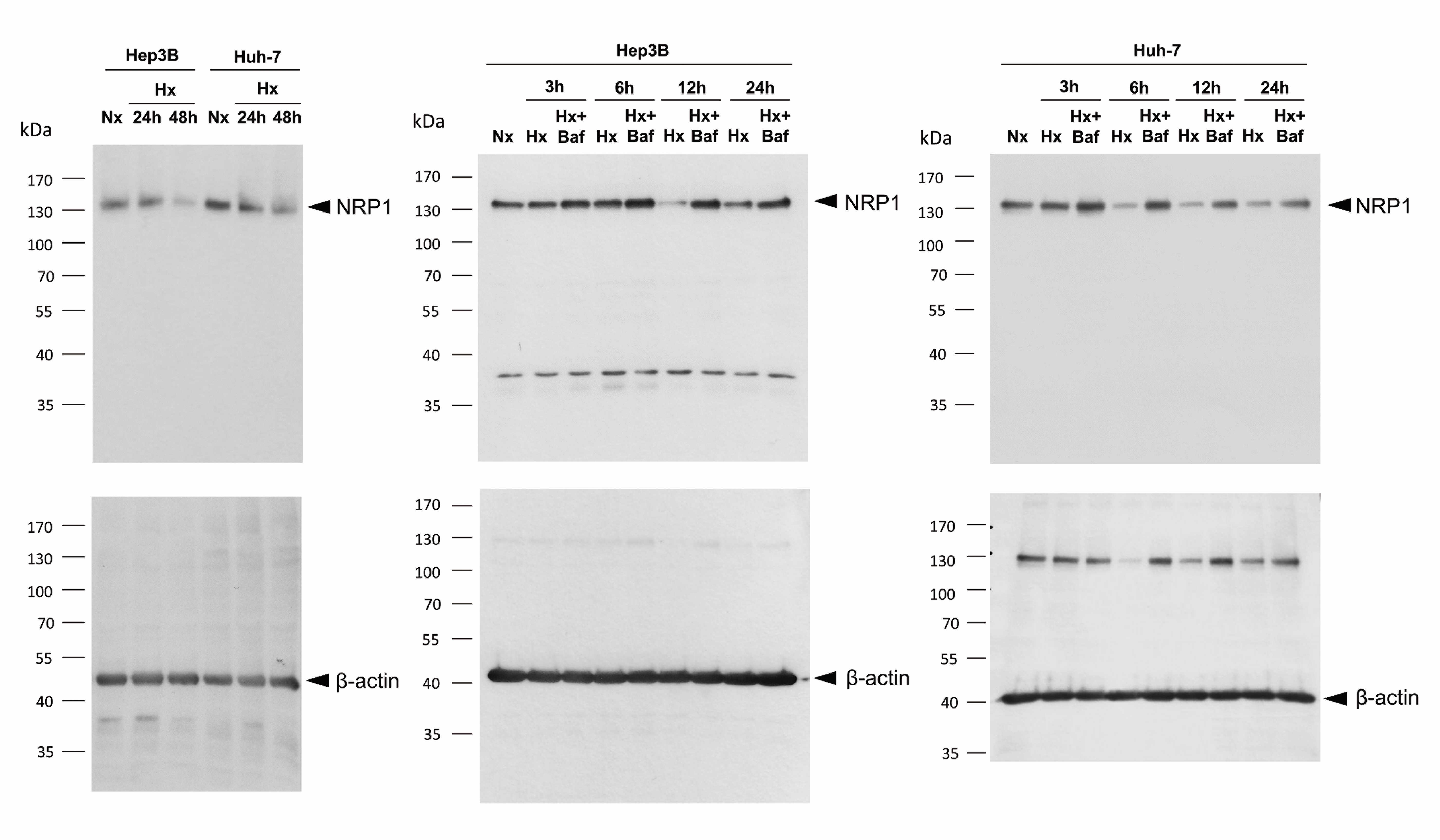

Supplement: Supplementary file 15 — Supplementary Fig. S15 [file 41401_2022_1021_MOESM15_ESM.jpg]

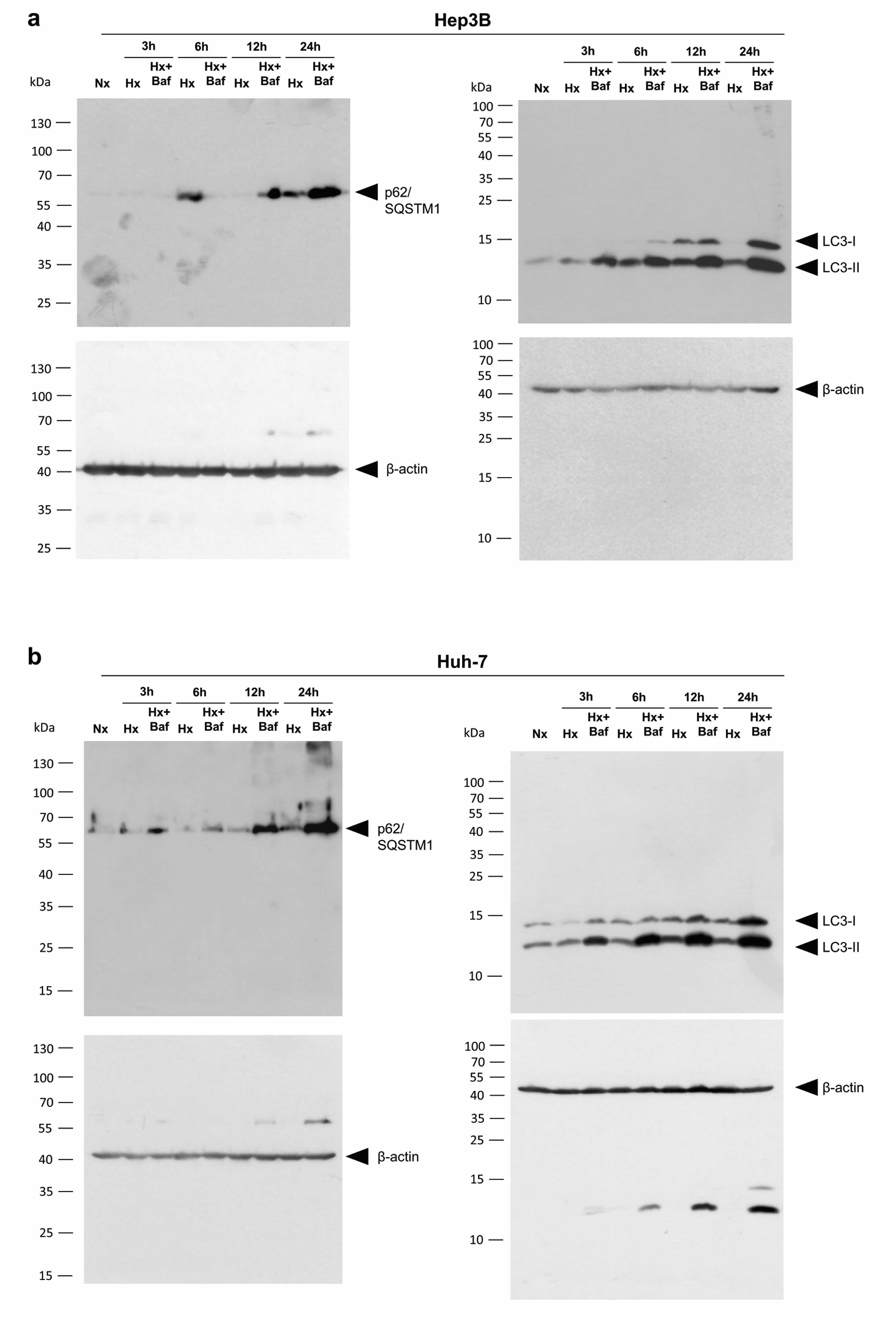

Supplement: Supplementary file 16 — Supplementary Fig. S16 [file 41401_2022_1021_MOESM16_ESM.jpg]

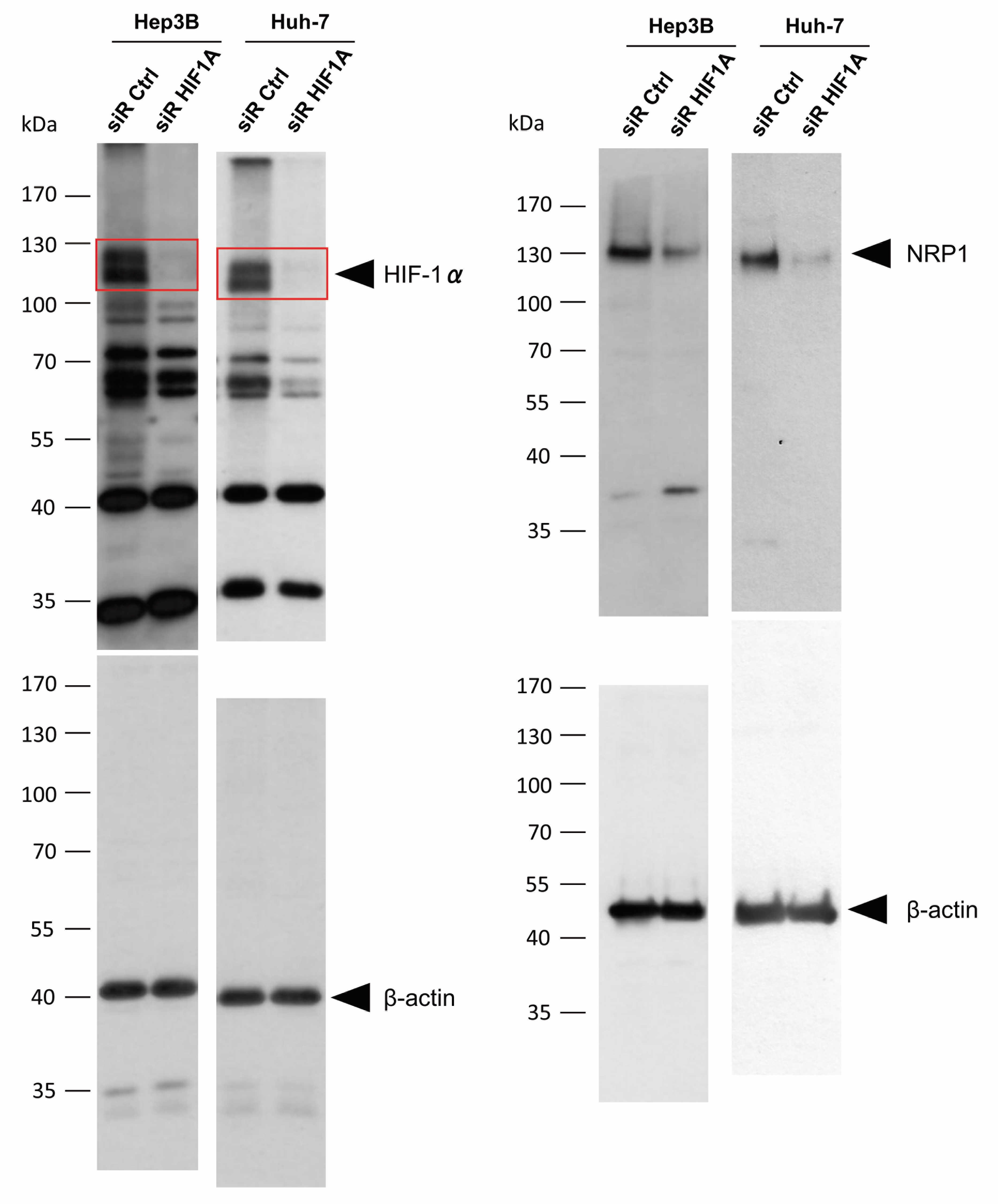

Supplement: Supplementary file 17 — Supplementary Fig. S17 [file 41401_2022_1021_MOESM17_ESM.jpg]

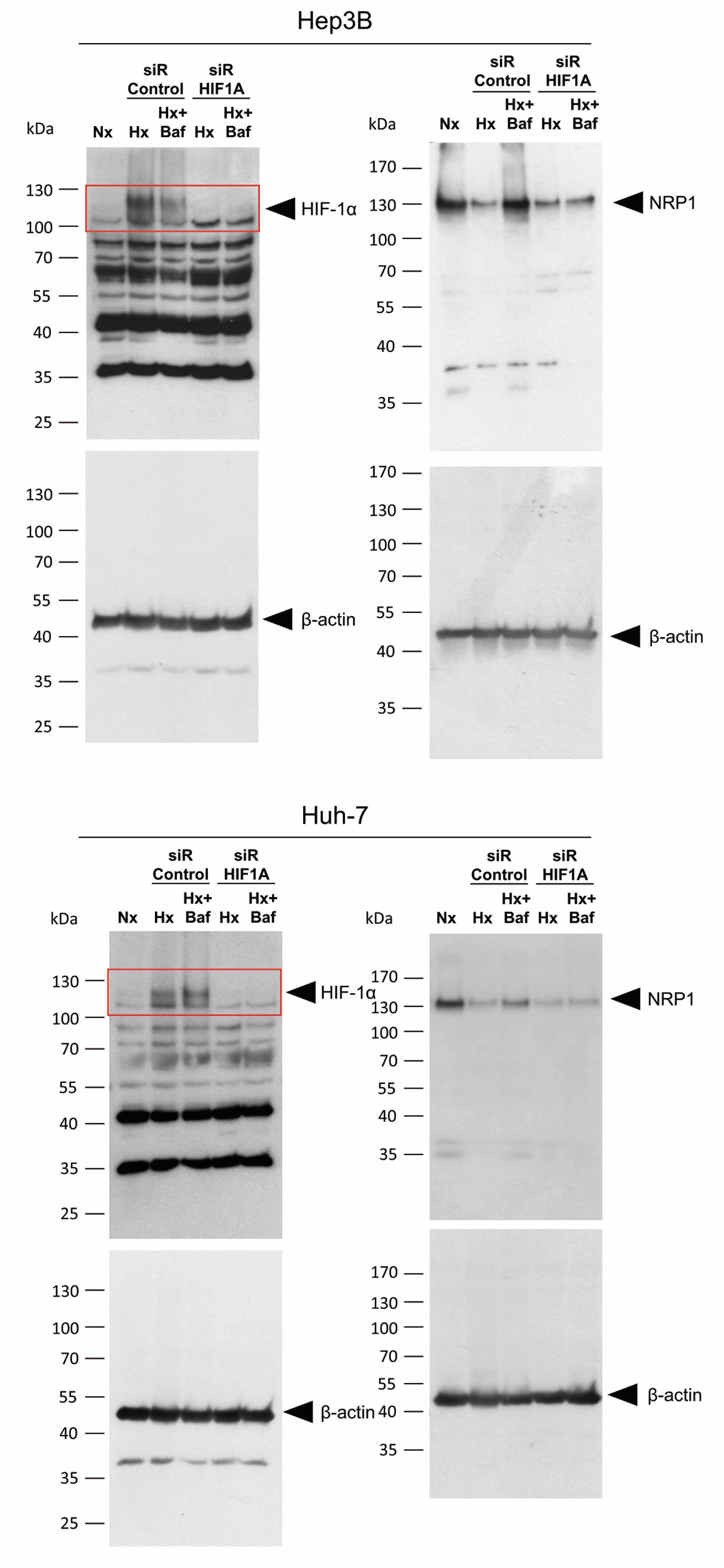

Supplement: Supplementary file 18 — Supplementary Fig. S18 [file 41401_2022_1021_MOESM18_ESM.jpg]

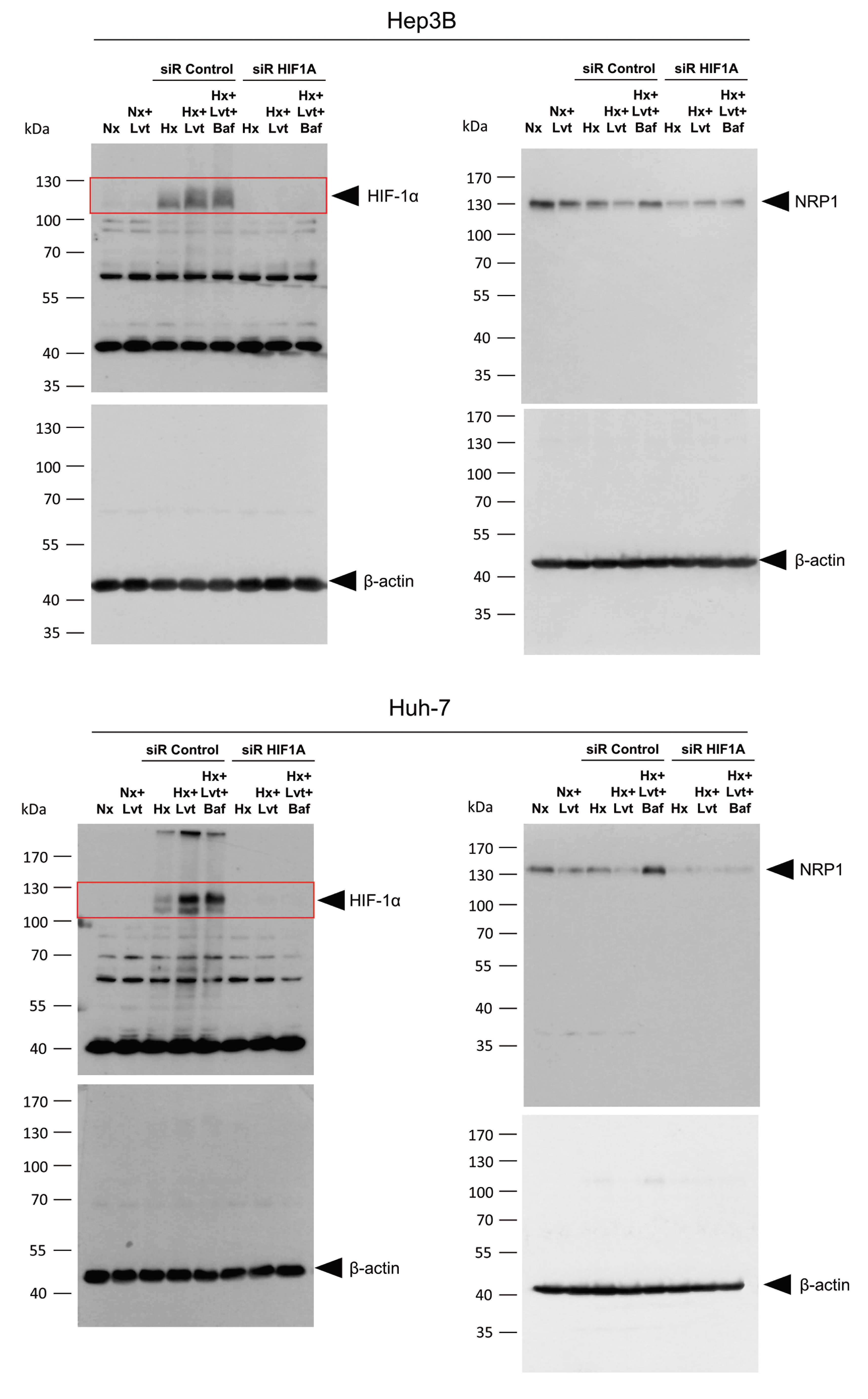

Supplement: Supplementary file 19 — Supplementary Fig. S19 [file 41401_2022_1021_MOESM19_ESM.jpg]
