# Supplementary material for: Hepatocellular carcinoma cells loss lenvatinib efficacy in vitro through autophagy and hypoxia response-derived neuropilin-1 degradation
Source: Acta Pharmacol Sin. 2022 Nov 14;44(5):1066–82. doi: 10.1038/s41401-022-01021-2 (PMC10104874; doi:10.1038/s41401-022-01021-2)
Supplement: Supplementary file 20 — Supplementary figure legends [file 41401_2022_1021_MOESM20_ESM.docx]

**Supplementary Fig. S1. Lenvatinib effects on ICC of Ki67 and NRP1 with split and merge confocal images.** (**a**) nuclear translocation of Ki67 and (**b**) total NRP1 after lenvatinib (Lvt) treatment for 48 h. Magnification 63×, scale bar 10 µm. Split and merge images from the Fig. 2c, f.

**Supplementary Fig. S2. Analysis of cell viability inhibition by EG00229.** Cell viability of both HCC cells lines Hep3B (upper panels) and Huh-7 (lower panels) was determined after 24 h and 48 h of EG00229 treatment with different concentrations. Data are represented as % of mean values relative to control ± SD (*n* = 5). **P*<0.05, ***P*<0.01, ****P*<0.001 *vs* control (non-treated cells).

**Supplementary Fig. S3. Effects of targeting NRP1 on lenvatinib actions on Ki67 proliferation index and NRP1 ICC expression with split and merge confocal images.** (**a**) Total NRP1 and (**b**) nuclear translocation of Ki67 after lenvatinib (Lvt) and/or EG00229 (EG) treatment for 24 h after another 24 h of NRP1 silencing (siR NRP1). Magnification 63×, scale bar 10 µm. Split and merge images from the Fig. 3b, e.

**Supplementary Fig. S4. Effects of targeting NRP1 on lenvatinib actions on cell migration ability.** Representative images from the wound-healing assay performed after lenvatinib (Lvt) and/or EG00229 (EG) treatment for 24 h after another 24 h of NRP1 silencing (siR NRP1). Microscope images corresponding to the quantifications from Fig. 3f. Magnification 10×, scale bar 50 µm.

**Supplementary Fig. S5. Effects derived from autophagy inhibition on NRP1 protein expression and Ki67 proliferation index with split and merge confocal images.** (**a**) Total NRP1 and (**b**) nuclear translocation of Ki67 after lenvatinib (Lvt) and/or bafilomycin A1 (Baf) treatment for 24 h after another 24 h of NRP1 silencing (siR NRP1). Magnification 63×, scale bar 10 µm. Split and merge images from the Fig. 5b, e.

**Supplementary Fig. S6. Effects derived from autophagy inhibition on lenvatinib actions on cell migration ability.** Representative images from the wound-healing assay performed after lenvatinib (Lvt) and/or Bafilomycin A1 (Baf) treatment for 24 h after another 24 h of NRP1 silencing (siR NRP1). Microscope images corresponding to the quantifications from Fig. 5f. Magnification 10×, scale bar 50 µm.

**Supplementary Fig. S7. Analysis of the modulation on NRP1 protein levels by an *in vitro* hypoxic microenvironment with split and merge confocal images.** Total NRP1 in normoxia (Nx) and after hypoxia induction for 24 h and 48 h. Magnification 63×, scale bar 10 µm. Split and merge images from the Fig. 6b.

**Supplementary Fig. S8. Analysis of NRP1 modulation by hypoxia conditions and autophagy blockade in the HepG2 cell line.** NRP1 expression was determined by Western blot in normoxia (Nx) and after hypoxia (Hx) induction and/or autophagy inhibition by treatment with 100 nM bafilomycin A1 (Baf). (a) Crop and (b) complete immunoblots, as well as the densitometry reading of each band relative to normoxia, are shown.

**Supplementary Fig. S9.** Full-length immunoblots from (**a**) Fig. 1h, (**b**) Fig. 2e and (**c**) Fig. 3a. PVDF membranes from (**b**) were precut for simultaneous antibody incubation.

**Supplementary Fig. S10.** Full-length immunoblots from Fig. 4a.

**Supplementary Fig. S11.** Full-length immunoblots from Fig. 4b.

**Supplementary Fig. S12.** Full-length immunoblots from Fig. 4d for the Hep3B cell line for (**a**) p62/SQSTM1 and (**b**) LC3 proteins.

**Supplementary Fig. S13.** Full-length immunoblots from Fig. 4d for the Huh-7 cell line for (**a**) 3 and 6 h, and (**b**) 12 and 24 h. PVDF membranes from (**a**) were precut for simultaneous antibody incubation.

**Supplementary Fig. S14.** Full-length immunoblots from Fig. 5a.

**Supplementary Fig. S15.** Full-length immunoblots from Figure 6a, c.

**Supplementary Fig. S16.** Full-length immunoblots from Figure 6e for the (**a**) Hep3B and (**b**) Huh-7 cell lines.

**Supplementary Fig. S17.** Full-length immunoblots from Figure 7d.

**Supplementary Fig. S18.** Full-length immunoblots from Figure 7e.

**Supplementary Fig. S19.** Full-length immunoblots from Figure 7g.
